# The Healthy Homes Study: Protocol for a cluster randomized trial of a place-based smoke-free home intervention in affordable housing

**DOI:** 10.1371/journal.pone.0328786

**Published:** 2025-07-29

**Authors:** Mark R. Hawes, Deepalika Chakravarty, Jing Cheng, Margaret A. Handley, Janice Y. Tsoh, Tracy Kuo Lin, Robert A. Hiatt, Maya Vijayaraghavan

**Affiliations:** 1 Center for Tobacco Control Research and Education and Benioff Housing and Homelessness Initiative, University of California San Francisco, San Francisco, California, United States of America; 2 Division of Prevention Science, University of California San Francisco, San Francisco, California, United States of America; 3 Division of Oral Epidemiology and Public Health and Division of Biostatistics, University of California San Francisco, San Francisco, California, United States of America; 4 Department of Epidemiology and Biostatistics, University of California San Francisco, San Francisco, California, United States of America; 5 Department of Psychiatry, University of California San Francisco, San Francisco, California, United States of America; 6 Institute of Health & Aging, University of California San Francisco, San Francisco, California, United States of America; 7 Division of General Internal Medicine, University of California San Francisco, San Francisco, California, United States of America; PLOS: Public Library of Science, UNITED STATES OF AMERICA

## Abstract

**Introduction:**

Comprehensive clean air policies reduce exposure to secondhand tobacco and cannabis smoke, as well as nicotine aerosols, and improve health outcomes. However, these policies often do not apply to the eight million residents of multiunit affordable housing, many of whom are from minoritized populations. One strategy to promote smoke-free living environments is to increase the voluntary adoption of no-smoking rules in the home.

**Methods:**

We describe the protocol for the Healthy Homes study—a wait-list cluster randomized controlled trial of a smoke-free home intervention for affordable housing residents. The intervention was adapted from a prior version using the Capability Opportunity Motivation-Behavior (COM-B) model and the Behavior Change Wheel. We will enroll 544 residents at 48 affordable housing sites across Northern California. Sites will be randomized to intervention or wait-list control. Resident participants will receive a one-hour coaching session on how to adopt a smoke-free home. Housing staff will be trained as lay health workers to provide brief cessation coaching. Residents and staff will complete follow-up visits at 3 and 6 months. The intervention will be delivered in English, Spanish, Chinese (Mandarin and Cantonese), and Vietnamese. The primary resident outcome is voluntary adoption of a smoke-free home for 90 days or more at 6 months. The secondary outcome is carbon monoxide–verified point prevalence tobacco abstinence. For lay health workers, the primary outcome is change in Smoking Knowledge, Attitudes, and Practices scores. We will assess cost-effectiveness and use the Consolidated Framework for Implementation Research to evaluate implementation outcomes, including characteristics of successful adopters and multilevel drivers of behavior change.

**Discussion:**

Expanding access to smoke-free affordable housing is critical for reducing racial and ethnic health inequities. This study has the potential to support voluntary smoke-free home adoption, increase quitting, and reduce secondhand smoke exposure among affordable housing residents.

**Trial registration:**

ClinicalTrials.gov NCT06170437

## Introduction

Cigarette smoking causes 480,000 deaths in the United States (US) each year, including 41,000 deaths attributable to secondhand smoke exposure (SHS) [1]. Estimates of e-cigarette and cannabis-related deaths are currently unknown, although e-cigarette vapors contain known carcinogens, and studies have found that cannabis use is associated with increased risk of cardiovascular disease, head, neck, and other cancers, and all-cause mortality [2–5]. Comprehensive clean air policies reduce exposure to secondhand tobacco, cannabis smoke, and nicotine aerosols (e.g., e-cigarettes) and improve health outcomes from tobacco-related cardiovascular disease, pulmonary disease, and cancer [6–8].

### Smoke-free policies in affordable housing

Although smoke-free policies have known public health benefits, these policies are often not implemented in private and public multiunit housing in the US, where over 80 million people live [9]. Smoke-free policies are particularly relevant to the 8 million people living in multiunit affordable housing. Affordable housing is defined as multiunit housing that is subsidized by the Department of Housing and Urban Development (HUD) and includes public housing authority housing, housing choice voucher programs (i.e., formerly Section 8 housing), permanent supportive housing, and other types of subsidized housing. The prevalence of tobacco use and exposure and its associated morbidity and mortality is high among residents in affordable housing, where minoritized communities are overrepresented and where neighborhood structural factors (e.g., tobacco retail density) contribute to high rates [10,11].

To address disparities in tobacco exposure, HUD implemented an indoor smoke-free policy in 2018 that applied to 3,200 multiunit public housing authority housing sites across the US [12,13]. Evaluations of this policy in public housing indicate that implementation and enforcement challenges have limited the impact of this approach [10,14,15]. Concerns around enforcement, including the use of punitive measures like eviction for policy violations and loss of market share in properties, are some of the identified barriers to implementing a smoke-free multiunit public housing policy [14–16]. Moreover, this policy did not apply to the majority of multiunit affordable housing (e.g., permanent supportive housing, privately managed subsidized housing) – where smoke-free policies are rare, and residents are vulnerable to tobacco exposure. While smoke-free policies in multiunit housing can reduce tobacco exposure, additional approaches are needed to increase access to smoke-free environments in affordable housing.

### Evidence-based voluntary approaches for smoke-free home adoption

One solution to filling the gap in access to smoke-free living environments, as well as improving the implementation of smoke-free policies, may be to increase the voluntary adoption of smoke-free homes (i.e., a voluntary no-smoking rule in one’s home). Smoke-free homes are an evidence-based approach associated with reduced exposure to secondhand smoke and increased tobacco cessation [17–21]. A smoke-free home intervention that included mailings and a coaching call to 211 Helpline callers showed increased voluntary smoke-free home rules compared to the control groups [17,20,22]. We piloted a smoke-free home intervention in permanent supportive housing for formerly homeless adults, aimed at both residents and staff, and found that the “ground up” approach that relied on resident buy-in was an effective way of promoting smoke-free environments even in places with smoke-free policies [23].

### Adaptation of the Healthy Homes Study protocol

The Healthy Homes study is a place-based intervention [24] designed and developed in partnership with housing authorities and will be delivered in affordable housing across Northern California (ClinicalTrials.gov ID: NCT06170437). We adapted the Healthy Homes study protocol from our previously developed smoke-free home intervention [25], using the Capability Opportunity Motivation-Behavior (COM-B) model and the Behavior Change Wheel (BCW) [26–28].

**Fig 1:**
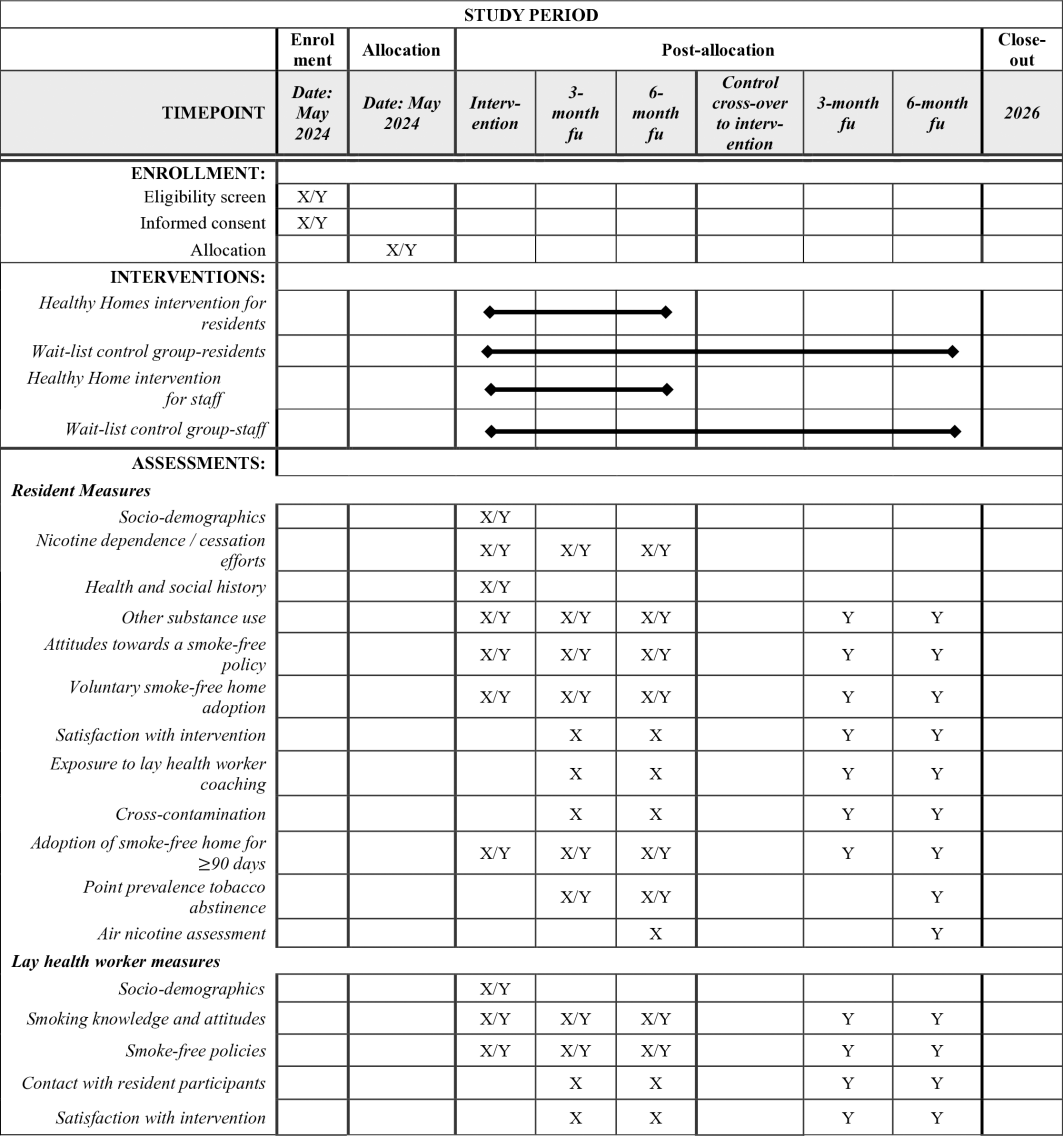
SPIRIT Flow Diagram: randomization, recruitment, intervention delivery, and assessment schedule. Legend: X indicates measure or action for the intervention group, Y indicates measure or action for the wait-list control group.

The Healthy Homes study is a multilevel intervention that (a) provides coaching to residents on how to adopt a smoke-free home and (b) trains housing staff as lay health workers to provide brief cessation coaching (education and guided support) to residents. Lay health workers are “insider messengers” who may share similar cultural and social backgrounds as residents and can deliver health messages to facilitate learning of new knowledge and provide peer support for behavior change [29–31]. Three housing authorities in Northern California, each providing housing to over 20,000 low-income residents, agreed to participate in the study. The intervention will be available in English, Spanish, Chinese (Mandarin and Cantonese), and Vietnamese (Fig 1).

### Objectives and aims

**Fig 2:**
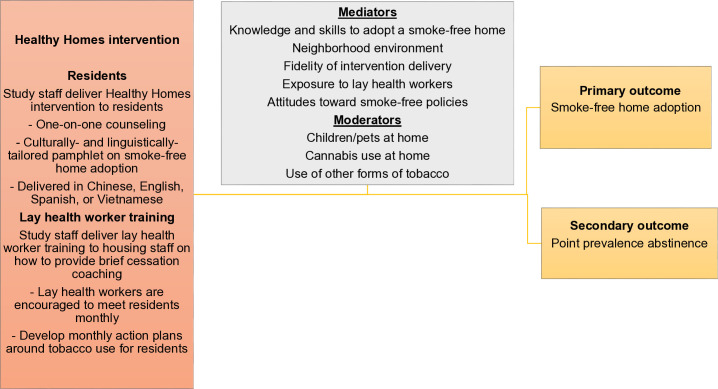
Overview of the adapted Healthy Homes intervention strategies, mediators, moderators, and outcomes.

The Healthy Homes study has three aims: (1) estimate the effect of the Healthy Homes intervention on affordable housing residents’ voluntary adoption of smoke-free homes; (2) determine the cost of the smoke-free home intervention and determine whether it is a cost-effective use of health care resources compared to usual care; (3) using the Consolidated Framework for Intervention Research (CFIR), evaluate implementation outcomes, including characteristics of successful adopters at the individual and site levels, and the extent to which the intervention mitigates individual, interpersonal, community, and societal levels of influences of tobacco use behavior. The intervention is expected to have the primary effect of increasing the adoption of smoke-free homes, and through the mechanism of adopting a smoke-free home, it may have a secondary effect of increasing tobacco cessation (Fig 2).

## Methods

### Study design

We will conduct a wait-list cluster randomized controlled trial in which housing sites will be randomized to intervention and wait-list control groups [25,32,33]. Participants in the intervention sites will receive the intervention first. Those in the wait-list control sites will receive usual care for the first 6 months and then be offered the choice to receive the intervention after they complete their 6-month follow-up visit; those in the wait-list control group who choose to receive the intervention will be observed for an additional 6 months. This study was approved by the University of California, San Francisco Protocol Review and Monitoring Committee (Institutional Review Board number: 23–39015).

### Theoretical foundation and intervention adaptation

The individually directed smoke-free homes intervention from which the current Healthy Homes study is adapted is based on the Social Cognitive Theory [34]. Our original smoke-free home intervention mapped to the following Social Cognitive Theory Constructs: behavioral capability (imparting knowledge/skills), reinforcements (internal/external reinforcements, incentivization), expectations (goal setting), and self-efficacy (materials to increase self-efficacy) [23]. It addressed the behavioral components of smoke-free home adoption by providing knowledge through the resident coaching intervention, boosting self-efficacy via skill-building through interactions with housing staff, and addressing environmental dimensions by integrating it within the context of participants’ homes [23,25].

**Fig 3:**
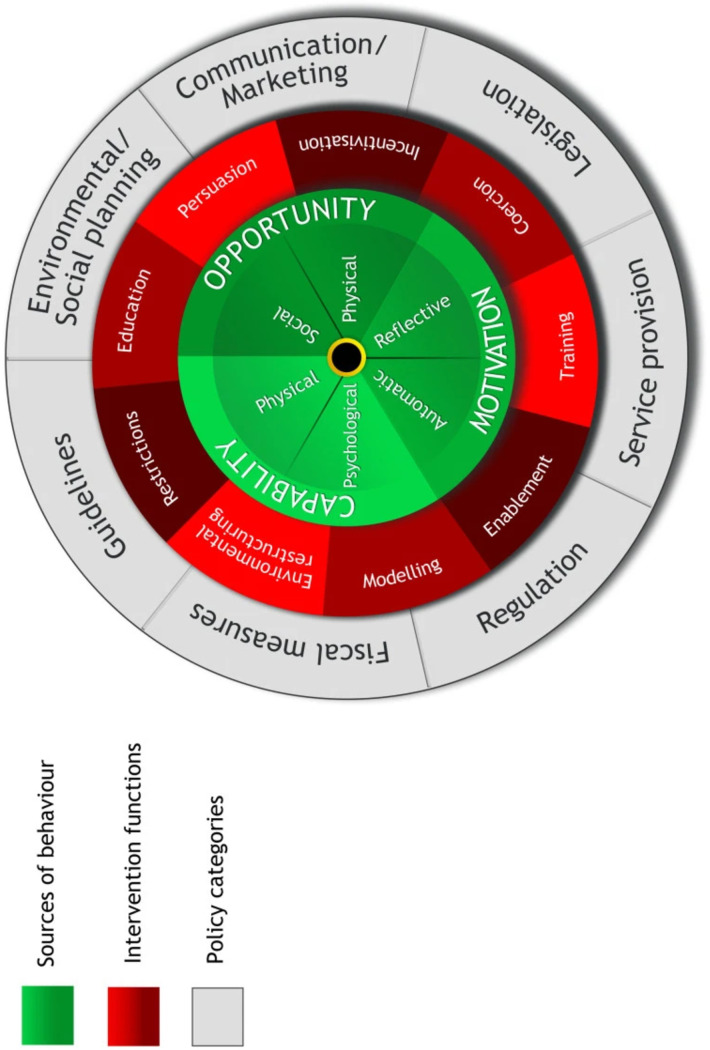
Behavior Change Wheel [27,35].

For the Healthy Homes study, we used the Behavior Change Wheel as a theoretical construct to conduct formative work [28] and to adapt the smoke-free home intervention for affordable housing. The Behavior Change Wheel comprises a comprehensive approach to behavior change and is widely applied to intervention development (Fig 3) [26,27,36].

The Behavior Change Wheel includes three layers: (1) the first uses the COM-B model (i.e., Capability (C), Opportunity (O), Motivation (M)-Behavior;) to highlight key barriers and potential enablers to voluntarily adopting a smoke-free home [26–28], (2) the second highlights eight possible intervention options that address the barriers identified in the COM-B domains; and (3) the third offers six policy options. The Behavior Change Wheel domains and corresponding elements of the Healthy Homes study are listed in Table 1.

**Table 1:** Mapping the Healthy Homes intervention delivery to the Behavior Change Wheel.

Barriers identified using Capability (C), Opportunity (O), Motivation (M)-Behavior (layer 1)	Intervention functions (Layer 2)	Policy targets (Layer 3)
**Resident barriers and enablers** - Decreased knowledge of tobacco, e-cigarette, and cannabis use and exposure (C) - Lack skills to adopt a smoke-free home (C) - Indoor cannabis use and co-use of other combustible tobacco or e-cigarettes interferes with smoke-free home adoption (C, M) - Neighborhood violence is a barrier to smoking outdoors (C) - Social norms support indoor tobacco use (M) Enabler - Lay health workers could support smoking cessation behaviors (O)	**Residents** - Education: Culturally-relevant information on tobacco, e-cigarette, and cannabis use and exposure - Training: Step-by-step guide to adopt a smoke-free home - Restrictions: Voluntary smoke-free home adoption - Persuasion: Smoke-free home pledges - Enablement: Monthly encounters with lay health workers on smoking cessation coaching	**Environmental or social planning** - Voluntary adoption of smoke-free homes - Exploring downstream effect of other residents in the building adopting smoke-free homes - Exploring community-level support for building level adoption of smoke-free policies **Service provision** - Lay health worker smoking cessation coaching - Referrals to smoking cessation services **Legislation** - Working with housing providers, tobacco control advocacy organizations, and city government to introduce ordinances around smoking in multi-unit housing
**Staff barriers** - Lack of skills to engage with residents on tobacco, e-cigarette, and cannabis use and exposure (C) - Lack of skills on conflict resolution around tobacco use (C) - Lack of skills on providing brief cessation coaching (O)	**Staff** - Training: Lay health worker training on how to provide coaching - Education: Delivering culturally-relevant coaching on smoking cessation, providing information on tobacco cessation resources - Enablement: Providing monthly action plans for tobacco cessation	

As part of the intervention adaptation process, we partnered with Section 8 Housing Choice Voucher affordable housing sites in San Francisco, California, and explored factors related to voluntarily adopting a smoke-free home among racially-ethnically and linguistically diverse populations [28]. We used the Behavior Change Wheel model (Fig 3) to identify barriers to and facilitators of the intervention, which allowed for the identification of needed modifications.

We made the following changes to our original evidenced-based smoke-free home intervention, using materials from the Centers for Disease Control and Prevention (CDC) and Kick It California [37–39]: (1) culturally and linguistically adapted content for monolingual Spanish, Chinese, and Vietnamese-speaking populations, 2) added content on e-cigarette and cannabis use and exposure; 3) added brief structured monthly lay health worker (i.e., trained housing staff) coaching sessions for residents to increase the rigor of the lay health worker coaching intervention component compared to ad-hoc coaching sessions in the original intervention (Table 1).

**Fig 4:**
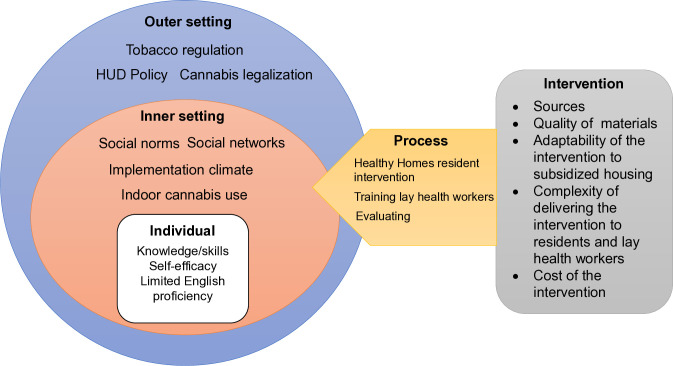
Consolidated Framework for Intervention Research (CFIR) framework for the adapted Healthy Homes Study.

We will use the Consolidated Framework for Implementation Research (CFIR) to evaluate implementation outcomes associated with Healthy Homes, including the intervention’s adaptability (i.e., can the intervention be adapted to meet local needs), scalability (i.e., ability to expand the intervention to other regions or settings), and sustainability (i.e., likelihood of continued use of the intervention to achieve the desired outcomes), and assess the characteristics of adopters at the individual and site levels [35,40]. The CFIR comprises five domains (1) Intervention refers to the intervention source, the quality of materials, and adaptability of the intervention; (2) the outer setting refers to external policy such as the HUD policy, county ordinances around smoking, legalization of cannabis; (3) the inner setting includes structural characteristics of the setting, social norms, and the implementation climate; (4) individual characteristics include knowledge and skills, self-efficacy, individual stage of change; and (5) the implementation process refers to planning, engaging, implementing, and evaluating the Healthy Homes intervention (Fig 4).

### Resident intervention

Bilingual study staff will use a language-concordant pamphlet to deliver a one-hour, one-on-one coaching session in Chinese, English, Spanish, or Vietnamese to resident participants at their housing site at the time of enrollment. This session includes the following intervention components mapped to the second level of the Behavior Change Wheel: 1) *Education* on tobacco, e-cigarette and cannabis use and exposure (secondhand and thirdhand smoke) for multigenerational households, and the personal cost of smoking; 2) *Training* on how to voluntarily adopt a smoke-free home; 3) *Restrictions* by encouraging voluntary adoption of a smoke-free home; 4) *Persuasion* using smoke-free home pledges; and 5) *Enablement* through monthly coaching interactions with lay health workers on smoking cessation (Table 1). We will observe 10% of the lay health worker and resident coaching sessions to assess the fidelity of the intervention using a checklist model and will make adjustments to the delivery of the intervention where there are substantial departures from the protocol.

### Staff lay health worker intervention

We will deliver the housing staff intervention in English using a lay health worker model using training, education, and enablement. Lay health workers who may be bilingual in English and Spanish, Chinese, or Vietnamese will receive a two-hour training on the principles of tobacco and nicotine addiction, interactions between tobacco, e-cigarette, and cannabis use, tobacco treatment approaches, including cessation coaching and pharmacotherapy, local resources for tobacco treatment, and talking points on tobacco cessation coaching in the different languages. Through the training, lay health workers will have a chance to model the 5As (Ask, Advise, Assess, Assist, and Arrange) for smoking cessation or 2As and R (Ask, Advise, and Refer), as well as initiate conversations around tobacco use with residents [23,25]. Bilingual lay health workers will be asked to engage with residents on a monthly schedule. Lay health workers will maintain a log of these interactions, including attempts to reach participants, and will complete a monthly assessment form that includes an assessment of readiness to quit and an action plan adapted from a family-based lay health worker-led smoking cessation intervention (31). The action plan will include prompts to (1) talk to family or friends, (2) make some changes towards smoke-free home adoption or smoking cessation, (3) call the Kick It California telephone quitline, and (4) talk to their healthcare provider or neighborhood pharmacy (if they do not have a healthcare provider) for cessation coaching. Because residents’ encounters with lay health workers will occur as part of routine care, we will request permission from a sample of staff and resident dyads (n = 40) to observe and record these interactions using a standardized evaluation form to assess fidelity.

### Randomization

The study will be conducted in 48 affordable housing sites across Northern California. We will use the method of restricted randomization—commonly applied in cluster-randomized trials and used in our previous trial—to ensure an acceptable level of balance across groups at baseline [25,33]. Our goal is to achieve balance for the following variables: property size (i.e., number of residents), smoking-related policies (e.g., existing smoke-free policies), and geography (e.g., location). First, we will create two sets of 24 housing sites, each balanced on these variables. Second, we will randomly assign one set of sites to the intervention and the other to wait-list control.

### Participants and setting

#### Resident participants.

We aim to recruit N = 544 resident participants, n = 272 each in the intervention and wait-list control arms. To be eligible, resident participants must: (1) identify as current smokers (smoked at least 100 cigarettes in their lifetime and smoke at least 5 cigarettes per day, verified by expired carbon monoxide [CO] ≥ 5 parts per million [ppm]) (41); (2) smoke in their home; (3) expect to live in the same housing site for at least 12 months; (4) be at least 18 years old; (5) speak Chinese, English, Spanish, or Vietnamese; (6) be able to meet with the study staff for followup visits; and (7) be able to provide informed consent. Exclusion criteria include a contraindication to study-related procedures or assessment (e.g., unable to provide informed consent).

#### Staff participants.

We expect to enroll 30–50 housing site staff to receive lay health worker training. Eligibility criteria are: (a) service or property management staff (e.g., case managers, counselors) who are at least 18 years old, (b) willing to engage in a two-hour lay health worker training, (c) bilingual in English and Chinese, Spanish or Vietnamese, and (d) able to provide informed consent. Housing staff at each site will be invited to participate via email. Study staff will screen housing staff interested in being lay health worker participants for eligibility and obtain written informed consent using the teach-to-goal method [41].

### Recruitment and enrollment

Study staff will contact participants through flyers in their mailboxes and in person at community engagement kickoff events to announce the study and gauge interest. During kickoff events, study staff will explain the study, inform residents and staff about the frequency with which they will be on-site for recruitment and follow-ups, and gather contact information from interested residents. Study staff will also do 1:1 in-person outreach by going door-to-door to inform residents about the study. Interested residents will be asked to sign up for eligibility screening, after which they will be enrolled in the study if they meet eligibility criteria. Lay health workers will be recruited via email and direct in-person outreach when study staff are on site. Study staff will be present at the housing sites during designated times to screen interested candidates for eligibility, obtain written informed consent using the teach-to-goal method [41], and enroll those eligible for the study. Study staff will conduct the training at a mutually agreed time.

### Control

Resident and staff participants in the wait-list control group will receive usual care, which involves no on-site treatment for smoking cessation or smoke-free home adoption.

### Study timeline and follow-up

The study duration for intervention group participants is 6 months from the time of enrollment. Wait-list control group participants (i.e., both residents and staff) will be offered the intervention after the intervention group participants within the same cluster receive their intervention; participants who choose to receive the intervention will be observed for an additional 6 months. Data will be collected for all participants at baseline (pre-intervention), 3 months, and 6 months; for the control cross-over set of participants, data will also be collected at 9 and 12 months (i.e., 3- and 6-months post-intervention) (Fig 1).

### Reimbursements

We will provide resident participants with gift cards for completing the baseline questionnaire ($20), 3-month questionnaire ($15), and 6-month questionnaire ($25). We will provide participants $5 gift cards for each monthly tracking visit between follow-ups. Participants selected for random air nicotine monitoring at 6 months will be reimbursed $5 for placing monitors in their homes for at least 7 days. Participants who complete all follow-ups and monthly check-ins will be offered an additional $50 at the 6-month follow-up. To support recruitment, participants will also receive a $10 gift card for referring eligible participants for up to 3 participants. These amounts have been used in prior studies with minimal risk of coercion [23,42,43]. We will provide lay health workers gift cards for the amount of $20 for completing the baseline questionnaire, $15 for completing the 3-month questionnaire, and $25 for completing the 6-month questionnaire. Lay health worker participants will receive an additional $5 gift card for completing the monthly engagement logs, and those who complete all surveys and follow-ups will receive an additional $50 at the 6-month follow-up.

### Retention

For each scheduled visit (i.e., 3- and 6-month follow-ups), we will attempt to contact participants at least three times up to a month after their visit due date before they are marked as having a missed visit. Between the scheduled quarterly assessments, we will conduct monthly incentivized check-ins (i.e., at 1, 2, 4, and 5 months) in person or by telephone to facilitate study retention. Based on data from our pilot study, we expect to have a retention rate of at least 75% at the 6-month follow-up [25].

### Data collection

We will record participant data using questionnaires on Research Electronic Data Capture (REDCap), a secure online data collection platform [44]. Study staff will administer the questionnaires in the preferred language of resident and lay health worker participants (Fig 1).

### Resident questionnaire measures

*Sociodemographics.* At baseline, we will obtain information on age, sex assigned at birth, gender identity, race/ethnicity, education, employment status, income from all sources, and length of stay in current residence.

*Social determinants of health.* We will obtain information on unmet needs [45], residential history [42,43,46], homelessness history [42,43,46], exposure to urban life stressors [47], quality of housing [25], and neighborhood safety [48,49].

*Neighborhood-level factors.* We will ask where participants obtained their tobacco products, and how far they have to travel to obtain tobacco, cannabis, and alcohol products [28].

*Alternative tobacco products and cannabis use.* We will assess lifetime and past 30-day use of e-cigarettes, cigars/little cigars, smokeless tobacco, hookah, blunts, and cannabis.

*Tobacco dependence and smoking cessation.* We will use Fagerstrom’s test for nicotine dependence [50], assess e-cigarette dependence [51], quit attempts (past year and in the past 3 months), length of the last quit attempt, and use of cessation aids [52].

*Smoke-free policy.* We will assess smoke incursion, knowledge of current no-smoking policies, past 7-day exposure to tobacco and cannabis, and attitudes toward smoke-free policies and indoor use of tobacco and cannabis [16,23,25,53].

*Measures related to voluntary smoke-free home adoption.* We will ask whether participants voluntarily adopted a smoke-free home in the past 90 days and the length of the last adoption, which will be used to define our primary outcome. Those who report a smoke-free home will be asked how many days they were smoke-free in their home in the past 90 days. Among those who report using cannabis indoors, we will explore whether participants voluntarily adopted a cannabis-free home and the length of time their homes were cannabis-free [16,23].

*Expenditures for tobacco use.* We will obtain information on the amount of money spent on tobacco in the prior week, which we will use to estimate tobacco-related expenditures [23].

*Chronic diseases.* Using the National Health Interview Survey, we will ask about diagnoses of liver disease, renal disease, heart disease, hypertension, diabetes, cancer, pulmonary disease, arthritis, hepatitis C and HIV [54].

*Mental health.* We will screen for depression using the 10-item Center for Epidemiologic Studies Depression Scale [55], anxiety using a seven-item anxiety scale (Generalized Anxiety Disorder-7) [56], and post-traumatic stress disorder (PTSD) using the Primary Care PTSD Screen [57].

*Alcohol and substance use disorders.* We will ask participants about lifetime use of opioids, methamphetamines, crack/cocaine, and cannabis, and for those who report lifetime use, we will ask about use in the past 30 days and number of days used in the past 30 days. To assess cannabis use, we will administer the Cannabis Use Disorders Identification Test-Revised (CUDIT-R) [58]. To assess risky alcohol use, we will administer the Alcohol Use Disorders Identification Test (AUDIT-C) [59,60].

*Satisfaction and usefulness of the intervention* For intervention recipients, at 3- and 6-month follow-ups, we will evaluate satisfaction with the intervention using questions with Likert responses and usefulness using a previously validated item, ‘In the past three months, what role did the intervention play in helping you quit smoking?’

*Exposure to lay health worker coaching* At the 3- and 6-month follow-ups, we will ask residents whether they had any coaching encounters with lay health workers, the number of these encounters, whether they were asked and advised to quit by lay health workers, and the satisfaction and usefulness of these encounters.

*Evaluating the potential for cross-contamination* At the 3- and 6-month follow-ups, we will ask participants who received the intervention whether they had discussed the intervention or shared materials with family and neighbors in their building.

*Cost data.* We will collect data on costs through publically available data (e.g., average sales price of cigarettes, CPT code for cessation services and cost, average time for cessation services) and study business ledger (e.g., intervention start-up material cost, training cost). We will collect and analyze lay health worker time use in the intervention through a time and motion study [61].

### Lay health worker questionnaire measures

*Sociodemographics.* At baseline, we will ask lay health worker participants to provide their age, gender identity, race/ethnicity, role in the facility, and smoking history (if applicable).

*Smoking knowledge and attitudes.* Lay health workers will complete the Smoking Knowledge Attitudes Practices (SKAP) survey at baseline, 3 and 6 months [62,63]. The SKAP survey assesses knowledge, attitudes, barriers, efficacy, and practices related to providing tobacco treatment [63].

*Smoke-free policies.* At baseline, we will ask lay health workers about policies around smoking at the site (e.g., policies restricting tobacco, indoor use of e-cigarettes and/or cannabis, provision of on-site cessation services), enforcement of smoke-free policies (complaints, warnings, or evictions), and barriers to enforcement of current policies.

*Contact with resident participants.* Lay health workers will complete a monthly assessment form that documents attempts to reach resident participants, an assessment of resident participants’ readiness to quit, and an action plan.

### Qualitative Interviews

We will use qualitative interviews to evaluate implementation outcomes of adaptability, scalability, and sustainability of the intervention mapped onto the CFIR domains. We will assess the degree to which the intervention was adopted across sites, how the intervention was implemented, and factors associated with scalability and sustainability [35,64]. We will: (a) identify characteristics of high and low adopters of the intervention at the individual and site levels; (b) examine whether there were differences in engagement in the intervention by language of administration of study components; (c) explore the adaptability of the intervention across subsidized housing sites including features of the intervention that did or did not work; (d) explore satisfaction with the intervention; (e) explore scalability of the intervention by leveraging bilingual housing staff to deliver the linguistically-tailored intervention; and (f) evaluate sustainability of the intervention including payment structures to support housing staff to adopt lay health worker roles.

### Outcomes and Analysis

#### Resident outcomes.

*Primary outcome.* The primary outcome is the percentage of participants with self-reported voluntary adoption of smoke-free homes for 90 days or more at the 6-month follow-up. We will ask participants to place a nicotine sampler in their units to better assess their environmental tobacco exposure at 6-months follow-up [17,65].

*Secondary outcome.* The secondary outcome is the percentage of participants with carbon monoxide verified point prevalence tobacco abstinence at the 6-month follow-up (expired carbon monoxide <5 ppm is defined as abstinence) [66].

#### Other outcome measures.

In addition, we will assess the percentage of participants adopting a cannabis-free home among cannabis users at a 6-month follow-up.

#### Lay health worker outcome.

The primary outcome of interest for lay health worker staff is a change in SKAP score from baseline to the 6-month follow-up.

#### Power and sample size.

The sample size calculation was based on the primary outcome of smoke-free home adoption of at least 90 days at the 6-month follow-up. We also conducted power calculations for the secondary outcome of point prevalence tobacco abstinence at the 6-month follow-up. Power analyses assumed 80% power; intra-cluster correlation of 0.01, two-tailed alpha = 0.05; and 75% retention at 6 months follow-up. With these parameters, we will need N = 544 residents, recruited from 48 housing sites to detect a group difference of 11.5% for the primary outcome of smoke-free home adoption, with 12% for the control arm versus 23.5% for the intervention arm (OR=2.25). For the secondary outcome of point prevalence abstinence at 6 months, the sample size provides 86.6% power to detect a group difference of 11% (13% versus 4%).

#### Statistical analyses.

We will estimate means, standard deviations, frequencies, proportions, and 95% confidence intervals for demographics (age, gender, race/ethnicity, education), other covariates (family size, health condition, insurance), and smoking-related variables (smoking history) at baseline by randomized groups. We will assess whether the variables are balanced at baseline by randomization (randomization check) and attrition (attrition analysis). Instead of using data from completed residents only, we will use all available data from all randomized residents in analyses while assuming that data are missing at random, conditional on observed variables. Multiple imputation will be used for sensitivity analyses with various assumptions on the missing mechanism [67–69].

Primary analysis will assess the intention-to-treat (ITT) effect of the intervention on adopting a smoke-free home compared to the control. The ITT approach will include all the randomized residents, including dropouts and noncompliers to the intervention, in the analysis. Specifically, a generalized linear mixed-effect model with logit link will be used to evaluate the group difference over time, with group (intervention vs. control), time (baseline, 3, and 6 months), and time*group interaction in fixed effects, and housing sites and residents in random effects (random intercept and/or slope). The corresponding group contrast at 6 months and its 95% confidence interval will be estimated. The 3-level data structure —including the housing site, residents, and repeated assessments — will be accommodated by the logistic mixed effects model with random intercepts for sites and residents.

We will conduct several secondary analyses. First, we will fit a generalized mixed-effects logistic model of the secondary outcome, point prevalence abstinence, similarly to the primary analysis. Second, participants lost to follow-up will be compared to those who completed the study to assess whether there are systematic differences in baseline covariates between the two groups. Third, we will include sex, race/ethnicity, and their interactions with the group and time in the logistic mixed effects model to examine any sex and/or race/ethnicity differences in the intervention effects on the outcomes. Fourth, we will perform mediation analyses to identify the mechanisms/pathways by which the intervention may have impacted smoke-free home adoption or point prevalence abstinence outcomes, including knowledge and skills to adopt a smoke-free home, change in smoke-free policy attitudes, exposure to lay health workers, neighborhood environment, and fidelity of intervention delivery. Fifth, we will explore the moderation effects of children or pets at home, indoor cannabis use, or other combustible tobacco use on intervention outcomes of smoke-free homes and point prevalence abstinence outcomes via mixed effects regression models. Replicability of within-group changes on primary and secondary outcomes will be tested by comparing baseline to 6-month changes in the intervention group to corresponding 6- to 12-month changes in the waitlist control group.

Analyses of lay health worker data will include a pre-post training analysis of the smoking knowledge, attitudes, practices, efficacy, and barriers scales (SKAP) using generalized mixed linear models. We will fit generalized mixed linear regression models to examine factors associated with a change in each of the scales, accounting for correlations due to clustering by sites and residents and adjusting for demographics, geographic location, lay health worker smoking status, coaching encounters with residents, and lay health worker job role.

#### Cost-effectiveness.

Upon completion of the intervention, a cost analysis will be conducted, and the direct cost of the study intervention will be determined using best practices for micro-costing [70] and methods from previous tobacco cessation trials [71–75]. In the case where the intervention is effective as hypothesized, we will conduct a cost-effectiveness analysis (using per quit and per smoke-free home as outcome variables) as well as a cost-utility analysis (using quality-adjusted life year as the outcome variable); incremental cost-effectiveness ratio will be presented. We will conduct our analyses aligning with trial-based economic evaluations [76] and present our methodology and assumptions following the Consolidated Health Economic Evaluation Reporting Standards (CHEERS) format for economic evaluations [77].

#### Qualitative Analysis.

We will audio-record interviews and transcribe recordings. These documents will be imported into the Atlas.ti.v9 qualitative analysis package to facilitate data management. We will analyze qualitative data using a directed content analysis approach [78], as we have done in prior studies [16,79–83]. Study staff will work with the PI to review transcripts and identify initial themes that are reflexive and interactive, and map to CFIR constructs. Each transcript will be ‘coded’ by labeling text segments with the appropriate theme. This process will be repeated for all domains of interest until all the transcript data have been given fine-grain codes. Study staff and the PI will resolve discrepancies in the assignment of codes through discussion and report a Cohen’s kappa for interrater reliability [16,79–83]. We will create a summary of findings (step 5) mapped to the CFIR domains.

### Current status

Recruitment began on July 12, 2024, and is ongoing at the time of manuscript submission. We anticipate completing recruitment by August 31, 2026, data collection by December 31, 2026, and expect to report results by December 31, 2027.

## Discussion

It is estimated that Americans spend 90% of their time indoors, and two-thirds of that time is spent at home [84]. Young children, people living with disabilities, and minoritized populations in the US are most likely to be exposed to housing hazards that can have a detrimental impact on health and equity [84]. While one of the important social determinants of health is access to housing, access to quality housing is equally important [85].

One of the most ubiquitous and modifiable characteristics of quality housing that impacts both health and racial equity is access to clean air, which is now even more limited with e-cigarette use and cannabis legalization in some states. Exposure to secondhand smoke increases as socioeconomic status decreases [86], and African American and Latino/x populations are disproportionately impacted [87]. For example, African American and Latino/x children are more likely to be exposed to secondhand smoke than white children [9,88]. Youth exposed to secondhand smoke are more likely to have poor academic performance than those unexposed [89]. Additionally, poor housing quality has been shown to increase respiratory, neurological, mental health, and cardiovascular conditions in adults and children [90,91].

The 2018 policy restricting indoor use of tobacco in multiunit public housing was an important policy with the potential for substantial impact on health outcomes, but it demonstrated the challenges of mandating smoke-free environments in multiunit housing. A study that looked at nicotine concentration levels pre- and post-policy implementation found no differences in nicotine levels [14], and a qualitative study among public housing residents and staff found numerous enforcement challenges and resistance to smoke-free policies [10]. Punitive consequences that include fines or the threat of evictions without access to tobacco treatment or approaches for resident buy-in further hinder efforts for successful implementation [92].

Smoke-free policy implementation in public housing demonstrates that top-down policies need to be supplemented with tailored approaches that gain resident buy-in and offer corresponding cessation support. When accompanied by supporting policies, health-promoting, place-based interventions that have the support of both residents and staff have a greater chance of sustainability and achieving desired outcomes of smoke-free home adoption and smoking cessation.

Resident buy-in can be achieved by providing voluntary place-based interventions that provide education, skill-building, and support through lay health worker staff and are developed in partnership with housing authorities. Lay health workers have established relationships with residents, which may make residents more likely to engage in conversations with them about smoke-free homes and smoking cessation [31]. Voluntary smoke-free programs offer a self-enforced approach that can overcome many of the implementation challenges experienced after the federal smoke-free mandates in public housing. Further, promoting voluntary adoption of smoke-free homes along with providing supportive cessation services increases rates of smoking cessation [23]. One potential limitation of our trial is that it is conducted in three Northern California counties, potentially limiting generalizability, though the housing authorities serve a large linguistically and ethnically diverse population.

Access to affordable, healthy housing is one of the most important ways to reduce racial/ethnic health inequities in the US. Improving access to Healthy Homes that are designed to promote access to clean air and exploring ways in which neighborhood characteristics influence tobacco use and cessation behaviors can provide us with the tools to reshape our neighborhoods and homes to promote health, well-being, and equity for all populations in the US.

## Supporting information

S1 File IRB protocol_healthyhomesprotocol_1.29.24_5.23.25_2025.07.11. (PDF)

S2 File SPIRIT_checklist_additional file_final. (DOCX)

S3 File Version 1_healthyhomes_protocol_2025.07.11. (PDF)

## Abbreviations

AUDIT-C: Alcohol Use Disorders Identification Test
2As and R: Ask, Advise, and Refer
5As: Ask, Advise, Assess, Assist, and Arrange
BCW: Behavior Change Wheel
CUDIT-R: Cannabis Use Disorders Identification Test-Revised
COM-B: Capability Opportunity Motivation-Behavior
CESD-10: Center for Epidemiologic Studies Depression Scale
CDC: Centers for Disease Control and Prevention
CFIR: Consolidated Framework for Intervention Research
HUD: Department of Housing and Urban Development
GAD-7: Generalized anxiety disorder-7
ITT: Intention-to-treat
PTSD: Post-traumatic stress disorder
RCT: Randomized control trial
SKAP: Smoking Knowledge Attitudes Practices
UCSF: University of California, San Francisco
