# Supplementary material for: The Healthy Homes Study: Protocol for a cluster randomized trial of a place-based smoke-free home intervention in affordable housing
Source: PLoS One. 2025 Jul 29;20(7):e0328786. doi: 10.1371/journal.pone.0328786 (PMC12306785; doi:10.1371/journal.pone.0328786)
Supplement: S3 File — (PDF) [file pone.0328786.s003.pdf]

Study Title

Protocol Number: CC #MVijayaraghavanC

Protocol Version Number: 1
Protocol Version Date: 11.7.23

Study Intervention: Healthy Homes Study: A protocol for a place-based smoke-free home intervention in federally subsidized housing

IND Number:

NCT Number:

Principal Investigator (Sponsor-Investigator)
Maya Vijayaraghavan, MD MAS
University of California, San Francisco
490 Illinois Street, #92C
San Francisco, CA 94143

Statistician
Jing Cheng

Revision History

Version 1.0 Date 11.7.23

Protocol Signature Page

1. I agree to follow this protocol version as approved by the Institutional Review Board (IRB).
2. I will conduct the study in accordance with Good Clinical Practices (ICH-GCP) and the applicable IRB, ethical, federal, state, and local regulatory requirements.
3. I certify that I, and the study staff, have received the required training to conduct this research protocol.
4. I agree to maintain adequate and accurate records in accordance with IRB policies and federal, state and local laws and regulations.

UCSF Principal Investigator

Maya Vijayaraghavan
____
Printed Name

______________________________ 11.7.23
Signature Date

Abstract

Title	A smoke-free home intervention in federally subsidized housing
Study Description	The smoke-free home intervention study aims to reduce racial/ethnic disparities in tobacco use and exposure by increasing access to smoke-free homes and cessation resources among racially/ethnically- and linguistically diverse populations in federally subsidized housing ("subsidized housing"). Black/African American, Hispanic/Latino, and Asian populations with limited English proficiency are over-represented in subsidized housing; these populations have higher rates of tobacco use and exposure than the general population. We have an ongoing smokefree home clinical trial in permanent supportive housing (PRMC ID 21631, NCT04855357), and the proposed clinical trial will be fashioned after this one with similar study design and procedures but different intervention components, study sites and study population. We will adapt our current smokefree home intervention in permanent supportive housing for formerly homeless adults to subsidized housing for racially/ethnically and linguistically diverse populations. We propose to conduct a wait-list cluster randomized controlled trial of the adapted smoke-free home intervention compared to usual care among N=544 residents from 24 subsidized housing sites in Contra Costa, Oakland, and San Francisco.
Study Intervention	The multi-faceted intervention, delivered by bilingual study staff in Chinese (Mandarin and Cantonese), English, Spanish, or Vietnamese includes: 1) one-on-one counseling to residents who are smokers on how to adopt a smoke-free home, and 2) training housing staff as lay health workers to deliver monthly brief cessation coaching to residents.

Study Population	There are no exclusion criteria based on gender, race, or ethnicity. About 30% of subsidized housing residents have limited English proficiency or are monolingual in Chinese, Spanish or Vietnamese. Estimates of targeted enrollment are based on the demographic distribution of Contra Costa, Oakland, and San Francisco Housing Authority resident population. We will select sites based on the property portfolio of each Housing Authority and identify sites where smoke-free policies don't exist or where some policies exist, but they are inconsistently enforced and residents smoke in their units. Our partners at the Contra Costa, Oakland and San Francisco Housing Authorities provided us demographic estimates of their resident population, which we use to plan our study sample. Of the resident population in San Francisco, 42% are African American, 26% are Asian, 18% are White, 10% are Hispanic/Latino, 1% are American Indian or Alaskan Native, 1% Native Hawaiian or Pacific Islander, and 3% are of mixed race and/or another race/ethnicity, and 50% are female. Of the resident population in Oakland, 54% are African American, 20% Asian, 10% Hispanic/Latinx, 12% White, 1% American Indian or Alaskan Native, 1% Native Hawaiian or Pacific Islander, 2% mixed race, and 50% female. Of the resident population in Contra Costa, 40% are African American, 12% Asian, 22% are White,19% Hispanic/Latinx, 3% American Indian or Alaskan Native, 2% are Native Hawaiian or Pacific Islander, and 2% are of mixed race and/or other race/ethnicity, and 50% female. We will use our existing data to inform targeted enrollment for transgender participants, estimating that approximately 4% of our sample to identify as transgender.
Primary Objective	The primary outcome is self-reported voluntary adoption of a smokefree home intervention for ≥90 days at 6-months follow-up.
Secondary Objectives	The secondary outcome is biochemically verified point prevalence abstinence at 6-months follow-up.

Recruitment Methods	We will recruit N=544 resident participants, with n=272 each in the intervention and wait-list control arms (~n=17 participants per site). Within each site, all resident participants will be informed about the study and invited to participate. We will recruit participants within blocks of four housing sites per month, with each block containing two intervention and two wait-list control sites and anticipating roll-out of one such block every 4 to 5 months. We anticipate completing recruitment and enrollment of all participants in 24 to 30 months, allowing for a one-to-two-month extension. All residents in the intervention sites will be informed about the study through flyers in their mailbox, in person community engagement “kick-off” events at the time of initial recruitment, and those interested will be asked to sign up for eligibility screening. Study staff will also conduct in-person outreach for recruitment. At each site, we estimate that n=60 residents will be approached for eligibility screening, n=30 will be eligible, and n=17 will enroll into the study. We expect to enroll 50 housing staff to receive lay health worker training (2 to 3 staff per site). We will offer the intervention to residents and housing staff in the wait-list control sites once all participants in the intervention sites from the same block have completed their 6-month follow-up.
Sample Size	The sample size calculation was based on the primary outcome of smoke-free home adoption ≥90 days at 6-months follow-up. We also conducted power calculations for the secondary outcome of point prevalence abstinence at 6-months follow-up. Power analyses assumed 80% power; intra-cluster correlation of 0.01, two-tailed alpha=0.05; and 75% retention at 6 months follow-up. With these parameters, we will need N=544 residents who smoke, recruited from 24 housing sites to detect a difference of 11.5% for the primary outcome of smoke-free home adoption, with 12% for the control arm versus 23.5% for the intervention arm (OR=2.25), which compares favorably with 31.3% obtained in the pilot study.[1] For the secondary outcome of point prevalence abstinence at 6 months, the sample size provides 86.6% power to detect a difference of 11% (13% versus 4%), which compares favorably with 16.9% obtained in the pilot.[1]
Duration of Study Participation	It will take 6 months for the intervention site participants to complete the study and 12 months for the wait-list control site participants to complete the study. It will take 6 months for the lay health workers in the intervention and control sites to complete their participation in the study.

Unique Aspects of this Study	• Our adapted intervention will be delivered in Chinese, English, Spanish, or Vietnamese to reach linguistically and racially/ethnically diverse populations in subsidized housing.<br>• The resident-endorsed approach of voluntary adoption of smoke-free homes offers a novel pathway to increase access to smoke-free living in housing where there is no mandated policy.<br>• If the intervention is found to be effective, we will generate preliminary data in support of an implementation and dissemination grant of the adapted smoke-free home intervention in other types of subsidized housing that lack policies regionally and nationally.

List of Abbreviations

Add/Remove abbreviations as applicable to the study protocol

AE	adverse event
CRF	case report form
CTCAE	Common Terminology Criteria for Adverse Events
CTMS	Clinical Trial Management System
DSMC	Data and Safety Monitoring Committee
DSMP	Data and Safety Monitoring Plan
GCP	Good Clinical Practice
HDFCCC	Helen Diller Family Comprehensive Cancer Center
HIPAA	Health Insurance Portability and Accountability Act
ICF	informed consent form
ICH	International Conference on Harmonization
IRB	Institutional Review Board
PRMC	Protocol Review and Monitoring Committee (UCSF)

Table of Contents

Protocol Signature Page 2
Protocol Signature Page – Participating Sites 4
Abstract 3
List of Abbreviations 7
Table of Contents 8
1 Introduction 11
1.1 Background on <<condition, symptom, behavior, or other primary study focus>> 11
1.2 Background on <<study intervention>> 12
1.3 Study Rationale 13
1.4 Risk/Benefit Assessment 13
2 Study Objectives and Endpoints 13
2.1 Primary Objective 16
2.2 Secondary Objective(s) 16
2.3 Exploratory Objective(s) 16
3 Study Design 17
3.1 Characteristics 17
3.2 Sample Size 17
3.3 Primary Completion 18
3.4 Study Completion 18
4 Selection and Enrollment of Participants 18
4.1 Eligibility Criteria 18
4.1.1 Inclusion Criteria 18
4.1.2 Exclusion Criteria 18
4.2 Recruitment Methods 19
4.3 Inclusion of Women and Minorities 21
4.3.1 Eligibility of Women and Minorities 21
4.3.2 Recruitment of Women and Minorities 21
4.4 Inclusion Across the Lifespan 17
4.4.1 Age Range of Participants 22
4.4.2 Study Design/Recruitment Considerations Related to Age Groups 22
4.5 Participant Registration 22
4.6 Randomization/Assignment to Intervention 22
4.7 Blinding 23
5 Study Intervention 23
5.1 Administration and/or Delivery of Study Intervention 23
5.2 Interventionist Training and Tracking 23
5.3 Modifications to Administration of the Intervention and/or Supportive Care 24
5.4 Adherence Assessment 24
5.5 Concomitant Therapy 24

Table of Contents

5.5.1 Allowed Therapy........24
5.5.2 Required Therapy........24
5.5.3 Prohibited Therapy........24
5.6 Participant Discontinuation/Withdrawal from the Study........25
5.7 Lost to Follow-up........25
6 Study Procedures and Assessments........25
6.1 Schedule of Activities........26
6.2 Study Procedures and Assessments........27
6.2.1 Screening Period / Visit -1 (Day -# to Day 1)........27
6.2.2 Study Intervention Period........27
6.2.3 End of Study Intervention / Visit X (Day # +/- #)........27
6.2.4 Follow-up........28
7 Reporting and Documentation of Results........28
7.1 Measures and Instruments........28
8 Adverse Events and Serious Adverse Events........28
8.1 Definition of Adverse Event........30
8.2 Definition of Serious Adverse Event........30
8.3 Classification of Adverse Events........30
8.3.1 Severity........30
8.3.2 Attribution........30
8.3.3 Expectedness........31
8.4 Adverse Events Monitoring........31
8.5 Follow up of Adverse Events........31
8.6 Documenting and Reporting of Adverse Events........31
9 Statistical Considerations........31
9.1 Sample Size Considerations........31
9.1.1 Sample Size and Power Estimate........31
9.1.2 Randomization and Blinding........31
9.1.3 Stratification Factors........32
9.1.4 Accrual Estimates........32
9.2 Interim Analyses and Stopping Rules........32
9.3 Statistical Analysis Plans........32
9.3.1 Analysis Populations........32
9.3.2 Primary Analysis (or Analysis of Primary Endpoints)........32
9.3.3 Secondary Analysis (or Analysis of Secondary Endpoints)........32
9.3.4 Exploratory/Correlative Analysis/Assessments........33
10 Study Management........33
10.1 Pre-study Documentation........33
10.2 Institutional Review Board Approval........33
10.3 Informed Consent........33
10.4 Changes in the Protocol........33
10.5 Case Report Forms (CRFs)........34

Table of Contents

10.6 Record Retention 34
10.7 Publications 34
10.8 Multicenter communication *(for multicenter studies only – remove this section if the study will only be conducted at UCSF)* 31
10.9 Regulatory Documentation (for multicenter studies only – remove this section if the study will only be conducted at UCSF) 31
11 Protection of Human Subjects *(for multicenter studies only – remove this section if the study will only be conducted at UCSF)* 31
11.1 Protection from Unnecessary Harm 31
11.2 Protection of Privacy 32
12 References 35

1 Introduction

1.1 Background

Disparities exist in tobacco use and exposure among racial/ethnic groups in the US. Asians have among the lowest prevalence of tobacco use (7%), but sub-groups including Chinese men with limited English proficiency (LEP) have higher rates (22% compared to 14%).[2,3] Tobacco exposure in Chinese populations with LEP is also higher than the general population (66% to 86%).[4] Approximately 17% of Black/African Americans (hereafter "Blacks") report current tobacco use, with rates being higher in men compared to women (20.9% versus 13.9%).[5] Tobacco exposure is high in Blacks with over 45% of adults and 66% of children reporting lifetime exposure to tobacco smoke.[6] Hispanic/Latinos (hereafter "Latinos") have an overall low prevalence of tobacco use compared to Whites (10% versus 16.6%), but rates among Cubans, Mexicans, Central Americans, and Puerto Ricans are 2-3 times higher (15% to 28%).[2] These populations are the focus of this proposal.

Tobacco-caused cardiovascular disease and cancer are the first and second leading causes of death in Asians, Blacks, and Latinos.[7] Cancers of the lung and bronchus are the leading causes of death among Asians.[8] Blacks have lower rates of smoking and are lighter smokers than Whites, yet they experience more difficulty with quitting and have higher morbidity and mortality from tobacco use.[9-11] Blacks account for 6% of the adult population, yet they account for 8% of the yearly smoking expenditures, 13% of the mortality costs associated with tobacco use, 13% of all tobacco-exposure deaths, and 19% of the productivity losses from tobacco use.[12,13] Latinos have lower access to care, are more likely to experience delays in diagnoses of tobacco-caused cancers, have higher rates of diabetes that is linked with smoking, and higher productivity losses from tobacco exposure.[13-15]

Comprehensive smoke-free policies reduce tobacco exposure, hospitalizations from acute myocardial infarctions and other heart and lung disease [16,17] **and lung cancer incidence.**[18] In 2018, HUD introduced an indoor smoke-free policy in 3200 public housing sites across the US, impacting 1.2 million households.[19,20] However, enforcement challenges in public housing have limited the impact of this policy.[21-23] Moreover, the policy does not apply to the majority of subsidized housing, where over 8 million residents currently have no access to smoke-free policies and are vulnerable to tobacco exposure. Despite these challenges, the implementation of smoke-free policies in public housing presents a unique opportunity to improve health equity by reducing tobacco use and exposure in one's home. Therefore, expanding the reach of such policies to all types of subsidized housing could substantially reduce racial/ethnic disparities related to tobacco use and exposure.

The legalization of recreational cannabis has made the enforcement of smoke-free policies in multi-unit housing challenging. Smoking is the most common form of cannabis use, and secondhand exposure to cannabis smoke is increasing among multi-unit housing residents, particularly among subsidized housing families.[24,25] Low-income and minority populations in multi-unit housing are more likely to experience secondhand cannabis exposure because of low enforcement of smoke-free policies within multi-unit housing.[26-28] There is a need to better understand how cannabis use influences smoke-free policies in subsidized housing.

A smoke-free home—a voluntary no smoking rule in one's home—is associated with

reduced exposure to tobacco smoke among non-smokers and reduced consumption, increased quit attempts, and reduced relapse to smoking among smokers.[29-32] In subsidized housing that lack policies around smoking, smoke-free homes could bridge the gap in access to smoke-free policies. In subsidized housing that have policies on indoor smoking, but where enforcement is a challenge,[21,22,33,34,35] a voluntary smoke-free home approach could be instrumental in augmenting implementation and enforcement of such policies at the building level. Smoke-free homes that are inclusive of cannabis may also reduce cannabis use and exposure in subsidized housing. In this proposal, we aim to test the effectiveness of our adapted smoke-free home intervention in low-income, multi-ethnic and linguistically-diverse populations in subsidized housing in Contra Costa, Oakland, and San Francisco. **The intervention will have the primary effect of increasing adoption of smoke-free homes, and through the mechanism of adopting a smoke-free home, it may have a secondary effect of increasing tobacco cessation, which will translate to improved tobacco-related health outcomes for low-income, multi-ethnic diverse populations in California.**

1.2 Background on study intervention

We developed a smoke-free home intervention and evaluated its feasibility in permanent supportive housing for formerly homeless adults.[1] *Single-arm trial of 100 participants.* The intervention delivered by study staff had two components: 1) coaching residents to adopt a smoke-free home, and 2) training housing staff to provide ad-hoc cessation coaching to residents. *Theoretical framework*. The intervention was based on the social cognitive theory and addressed the behavioral components of smoke-free home adoption by providing knowledge through the resident coaching intervention and boosted self-efficacy via skill-building through interactions with housing staff, and addressed the environmental dimensions by integrating it within the context of participants' homes. *Findings from the pilot study.* At 6-months follow-up, 31.3% had voluntarily adopted a smoke-free home for ≥90 days, and 16.9% had carbon monoxide (CO)-verified point prevalence abstinence (PPA). Housing staff reported an increase in efficacy in delivering cessation coaching.[1] *RCT of the intervention.* In an ongoing RCT, we are evaluating the efficacy of the smoke-free home intervention in formerly homeless adults with mental health and substance use disorders in 20 permanent supportive housing sites.[36] The intervention is delivered in English by study staff in an in-person, face-to-face format (~ 30 minute session) using a smoke-free home pamphlet. While these preliminary results are promising for residents in permanent supportive housing, we learned that adaptations may be necessary to translate the intervention for linguistically-diverse populations in subsidized housing. Through our pilot and ongoing RCT, we learned that structured and brief monthly coaching sessions between residents and housing staff are feasible and will increase rigor of the housing staff coaching intervention over the ad-hoc sessions that are offered in the intervention in permanent supportive housing.

The adapted smoke-free home intervention builds on our previous model of in-person coaching for populations that have experienced homelessness[1,36] **and other models of telephone coaching for English-speaking low-income populations.**[37] We will modify the content and delivery of the adapted smoke-free home intervention in the following ways:

Theoretical framework. While the individually focused intervention is based on the social cognitive theory, to translate the intervention to subsidized housing and to lend rigor and structure to intervention adaptation, we mapped intervention functions to the Behavior Change Wheel. The Behavior Change Wheel is a theoretical framework that describes barriers to and

enablers of the behavior using COM-B (see above) and offers intervention functions and policy options to affect behavior change.[38-40] We adapted the intervention using the following intervention options: *Education* on the harms of tobacco use, *Training* on how to adopt a smoke-free home, *Restrictions* through voluntary smoke-free home adoption, *Persuasion* through the use of smoke-free home pledges, *and Enablement* through coaching interactions with housing staff. Policy options include increasing service provision for tobacco treatment and environmental or social planning through voluntary restrictions on smoking.

Delivery. We will deliver the 30-minute resident intervention in Chinese (Mandarin and Cantonese), English, Spanish, and Vietnamese using a culturally- and linguistically-tailored through in person interaction and a pamphlet. We will deliver the housing staff intervention using a lay health worker (LHW) model. We will train housing staff as LHWs, and we have incorporated structured monthly coaching sessions that include assessments and action plans. LHWs may share similar cultural and social background as residents, can provide knowledge as well as peer support for behavior change.[41-44] Housing staff in sites that serve monolingual residents are bilingual, have established a therapeutic alliance with residents, and meet residents monthly. These interactions could serve as a springboard for conversations on tobacco use.

1.3 Study Rationale

- Few interventions have focused on environmental exposure to tobacco in housing as a cause of tobacco-related health and cancer disparities. This adapted smoke-free home intervention is uniquely poised to reduce tobacco use and exposure in subsidized housing, thereby improving health equity for low-income, multi-ethnic populations in subsidized housing.
- This research will advance the field of tobacco control policies by: (1) examining whether resident endorsed and place-based interventions to reduce tobacco use and exposure might increase adoption of smoke-free policies in subsidized housing with and without county-mandated policies in multiunit housing; and (2) examining whether the implementation of policies are associated with reduction of smoking behaviors and secondhand smoke exposure.
- If the resident endorsed approach is effective and cost-effective in increasing voluntary adoption of smoke-free policies and ultimately smoking cessation, then it can be integrated into the implementation plans for cities and counties regionally and nationally that are considering smoke-free policies in their multiunit housing.

1.4 Risk/Benefit Assessment

Potential risks: There may be a potential for loss of privacy relating to the information asked in the questionnaires, particularly around issues on mental health disorders, substance use disorders, and previous history of homelessness. Loss of privacy or confidentiality – particularly around mental health, illicit substance use, and HIV status – could result in embarrassment or social marginalization. In the case of illicit substance use, loss of privacy or confidentiality could result in legal consequences. Because we will ask participants to consent to keep on file their name and contact and tracking information, there may be a potential for loss of confidentiality. Participants may experience symptoms of nicotine withdrawal from reducing consumption or quitting smoking. These symptoms may include irritability, fatigue, headaches or cravings for

cigarettes. Participants may also experience hunger as a result of reducing consumption or quitting smoking. There is a low risk of study participants experiencing fatigue and/or boredom during study visits. In addition to the above, we note other risks that may be relevant to our study population. Given the significantly low economic status of our study participants, study incentives may be coercive. We believe that the risk of these events is low given the measures we will put in place to minimize these risks (see below).

Adequacy of protection against risks

1. Procedures for protecting against loss of privacy. Although we collect basic information from subsidized housing residents and staff participants on socio-demographics and factors that can influence smoking cessation such as mental health disorders or substance use disorders, it will be kept to a minimum to minimize any psychological risk. All interviews will be conducted in a way that is formative without breaching the confidentiality of individuals participating in the study. We will ensure participants that participation in the study or responses to the questionnaire or in-depth, semi-structured interviews will not affect their eligibility for receiving housing services. We will assure participants who decide not to participate that their decision would not affect their ability to seek services. Participation in this study is voluntary.
2. Procedures for protecting against loss of confidentiality. There is a potential for loss of confidentiality, however we will take measures to minimize this risk. Each participant will have a unique arbitrary ID number. Identifying information collected from participants for tracking purposes will be stored separately from research data and will not be associated with the participants' unique study ID number. 'Talking Tablet Touchscreen Computers' will be used for data collection, and all data will be secured in an online database that is password protected. In-depth, semi-structured interviews will be audio-recorded. These recordings and their transcriptions will be stored on our secure server at UCSF. The server where data will be stored is backed up nightly. We will periodically go through the study data to make sure there is no personal identifying information. Only study staff will have access to study data. For added protection of confidentiality, we will apply for a Certificate of Confidentiality from the National Institute of Health.
3. Procedures for protection against legal risk to participants. The questionnaires will include questions about substance use behaviors (e.g., the use of illicit drugs). Procedures explained above for protecting privacy of individuals and confidentiality of data will minimize the legal risk to participants. For added protection of confidentiality of data, we will apply for a Certificate of Confidentiality from the National Institute of Health. Trained study staff will inform participants of legal risks during the informed consent process and will explain that participants can skip out of any interview questions they are not comfortable answering.
4. Procedures for protecting against coercion. Reimbursements may be coercive. There is controversy around whether reimbursements could be coercive or used to purchase illicit drugs when given to indigent populations participating in research. In trying to balance the need to reimburse participants for their time while limiting coercion and/or dangerous behaviors, we have set reimbursement levels comparable to those used in studies in similar

populations.[1,45-48] An alternative to cash reimbursements, we have chosen to reimburse in the form of gift cards for grocery stores and general pharmacies.

5. Procedures for protecting against hunger. It is possible that individuals who are attempting to quit smoking may experience hunger. Should this issue arise during the intervention, we will provide resident participants a list of resources of free food including food banks for food in bulk, soup kitchens for daily meals, and other free meal programs.
6. Procedures for protecting against withdrawal symptoms. Participants may experience symptoms of nicotine withdrawal from reducing consumption or quitting smoking. These symptoms include irritability, fatigue, headaches or cravings for cigarettes. LHWs will refer participants to their primary care providers as part of their coaching intervention. Participants who experience these symptoms will be referred to their primary care providers to obtain a prescription for nicotine replacement therapy or other medications for cessation and/or be evaluated for contraindications to these medications. Study staff will also ask participants about these symptoms during follow-up visits. If participants do not have primary care providers, we will refer them to local neighborhood health centers and/or pharmacies that can provide prescriptions for nicotine replacement therapy.
7. Procedures for handling reportable conditions. During the informed consent process, participants will be informed of information that must be reported by law should it be revealed during study visits, including suicidality and homicidality. The PI will train study staff on how to assess for symptoms of suicidality or homicidality, and the procedures to follow in the event of these occurrences. In addition, the PI will be always on call and will be available for consultation.
8. Procedures for protecting against fatigue or boredom. The enrollment study visit that will include completing a baseline questionnaire and viewing the video will last approximately 1.5 hours, and the follow-up visits at 3- and 6-months (intervention and wait-list control participants) and 9- and 12-months (wait-list control participants) will last approximately 30 minutes. The in-depth, semi-structured interviews in Aim 2 will last 60 minutes. Study staff will inform participants, during informed consent and at each study visit, that they can take breaks or stop participating in the study at any time.
9. Added protections for vulnerable populations. If a participant becomes incarcerated during the study, they will be censored from participation during incarceration. If released from jail/prison during the study time frame they can choose to re-enroll and a research assistant will repeat the informed consent process using procedures explained above.
10. Protections against COVID-19. We will ensure that all study procedures comply with current county-level protocols in place to minimize the impact of COVID-19. These procedure will include minimizing the number of days we are on-site to conduct recruitment, maintaining physical distancing and masking protocols, incorporating a universal COVID-19 screen at each encounter, and conducting follow-up visits by zoom or telephone in the event a participant has moved away. For participants, who screen positive for COVID-19 symptoms, we will refer participants to their primary care provider/medical homes to get tested and evaluated. For participants who report other needs because of COVID-19 (e.g., vaccinations, food, social services), we will request permission to share this information with their case managers and also refer them to their medical homes to get additional support.

Moreover, all staff will be required to wear personal protective equipment that adheres to state and local County guidelines. All participants will be required to wear a mask when interacting with study staff and maintain physical distance. If participants do not have a mask, study staff will provide one.

2 Study Objectives and Endpoints

2.1 Primary Objective

Primary Objective	Endpoint(s)	Time Frame
1. To assess whether the smoke-free home intervention is associated with self-reported voluntary adoption of smoke-free homes for ≥90 days at 6 months follow-up.	• Proportion of people who adopt a smoke-free home voluntarily	from time of recruitment to 6-months follow-up

2.2 Secondary Objective(s)

Secondary Objective	Endpoint(s)	Time Frame
1. To assess whether the smoke-free home intervention is associated with biochemically verified point prevalence abstinence.	• Proportion of people who achieve point prevalence abstinence	from time of enrollment to 6-months follow-up
2. To assess whether participating in the smoke-free home study is associated with change in behavioral outcomes for staff	• Continuous outcome measure of staff smoking knowledge, attitudes, practices, efficacy and barriers scales	From time of enrollment to 3-months follow-up and time of enrollment to 6-months follow-up

2.3 Exploratory Objective(s)

Exploratory Objective	Endpoint(s)
1. To assess voluntary adoption of cannabis free home among cannabis users	From time of enrollment to 6-months follow-up
2.	
3.	

3 Study Design

3.1 Characteristics

Pre-testing the adapted intervention. The multi-faceted intervention, delivered by bilingual study staff in Chinese (Mandarin and Cantonese), English, Spanish, and Vietnamese includes: 1) one-on-one counseling to residents who are smokers on how to adopt a smoke-free home, and 2) training housing staff as lay health workers to deliver cessation coaching monthly. In the first 9 months, we will: a) pre-test adapted intervention pamphlet with our bilingual study staff, residents and lay health workers, and the study's co-investigators using focus groups and in-depth interviews to ensure that translations are culturally relevant, b) obtain feedback from the Community Advisory Board on the intervention pamphlet and procedures, c) and set up the infrastructure for conducting the RCT including randomization scheme, recruitment strategies, and roll-out of the intervention.

Trial design. We will conduct a wait-list cluster RCT. Participants in the intervention sites will receive the intervention first. Those in the wait-list control sites will receive usual care and then cross over to the intervention arm after the intervention participants complete their 6-months follow-up.

Setting. Study procedures will take place in a private community room at the subsidized housing sites.

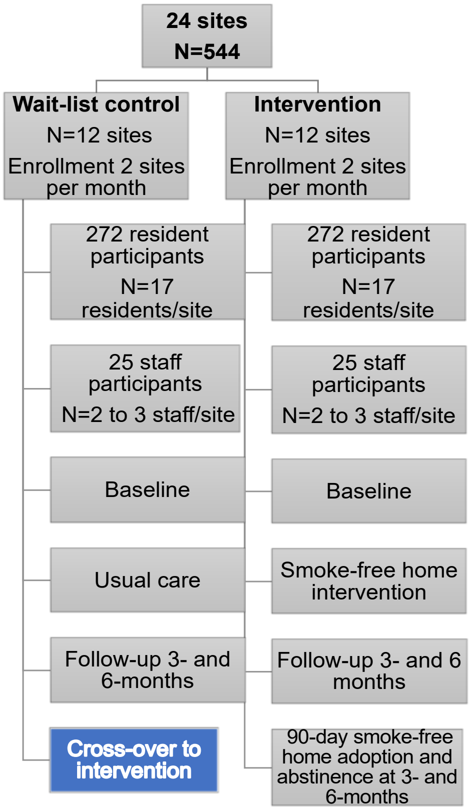

Figure 5: Randomization, recruitment, intervention delivery, and assessment schedule

Study procedures for resident participants. Eligible resident participants include current smokers (smoked at least 100 cigarettes in lifetime, daily or non-daily smoking in the past 7 days and at least 5 cigarettes per day, verified by expired CO ≥ 5 parts per million [ppm])[49,50] who expect to live in the subsidized housing site for at least 12 months, are ≥ 18 years of age, speak Chinese, English, Spanish, or Vietnamese and are able to provide informed consent. There are no exclusion criteria. Study staff will be present at the recruitment sites during designated times to screen interested participants for eligibility, obtain written informed consent using the teach-to-goal method,[51] and enroll those eligible into the study.

Study procedures for housing staff participants. Eligible housing staff participants will include service staff (e.g., case managers, counselors), who are ≥ 18 years of age, are willing to engage in a 2-hour lay health worker training and are able to provide informed consent. Staff in each site will be invited to participate, via email. Study staff will screen housing staff for eligibility, obtain written informed consent using the teach-to-goal method,[51] and offer dates to participate in the lay health worker training.

3.2 Sample Size

We will recruit N=544 resident participants, with n=272 each in the intervention and wait-list control arms. At each site, we estimate that n=60 residents will be approached for eligibility

screening, n=30 will be eligible, and n=20 will enroll into the study. We expect to enroll 50 housing staff to receive lay health worker training (2 to 3 staff per site). We will offer the intervention to the residents and housing staff in the wait-list control sites once all participants in the intervention sites from the same block have completed their 6-month follow-up. We will invite staff to enroll in the LHW training via email and expect to enroll about 30-50 housing staff (2 to 3 per site).

3.3 Primary Completion

The expected primary completion date is 36 months after the study opens to accrual. We expect to start data collection in March 1, 2024, and expect to complete all data collection by December 31, 2026 (36 months. after data collection starts, anticipated).

3.4 Study Completion

The expected study completion date is 40 months after the study opens to accrual, which is August 31, 2027 (anticipated)

4 Selection and Enrollment of Participants

4.1 Eligibility Criteria

4.1.1 Inclusion Criteria

In order to be eligible to participate in this study, an individual must meet all of the following criteria:

1. Age 18 years or older
2. Able to understand study procedures and to comply with them for the entire length of the study.
3. Ability of individual or legal guardian/representative to understand a written informed consent document, and the willingness to sign it.
5. Eligible resident participants include current smokers (smoked at least 100 cigarettes in lifetime, daily or non-daily smoking in the past 7 days and at least 5 cigarettes per day, verified by expired CO ≥ 5 parts per million [ppm])[49,50] who smoke in their homes.
6. Eligible housing staff participants will include service staff (e.g., case managers, counselors), who are ≥ 18 years of age, are willing to engage in a 2-hour lay health worker training, and are able to provide informed consent.
7. Expect to live and/or work in the subsidized housing site for at least 12 months, and
8. Speak Chinese, English, Spanish, or Vietnamese.

4.1.2 Exclusion Criteria

An individual who meets any of the following criteria will be excluded from participation in this study:

1. Contraindication to any study-related procedure or assessment.

4.2 Recruitment Methods

Recruitment schedule. We will recruit N=544 resident participants, with n=272 each in the intervention and wait-list control arms (~n=17 participants per site). Within each site, all resident participants will be informed about the study and invited to participate. We will recruit participants within blocks of four housing sites per month, with each block containing two intervention and two wait-list control sites and anticipating roll-out of one such block every 4 to 5 months. We anticipate completing recruitment and enrollment of all participants in 24 to 30 months, allowing for a one-to-two-month extension. At each site, we estimate that n=60 residents will be approached for eligibility screening, n=30 will be eligible, and n=17 will enroll into the study. We expect to enroll 50 housing staff to receive lay health worker training (2 to 3 staff per site). We will offer the intervention to residents and housing staff in the wait-list control sites once all participants in the intervention sites from the same block have completed their 6-month follow-up. We may add additional sites if we are not meeting our recruitment target.

As in our ongoing RCT in permanent supportive housing, we will recruit participants from subsidized housing sites across the San Francisco Bay Area, focusing on Contra Costa, Alameda (Oakland) and San Francisco counties. We will identify sites based on the Housing authorities' property portfolio and prioritize sites that don't have any smoke-free policies or where some policies may exist but they are inconsistently enforced and residents smoke in their units. Once we have contacted directors of each subsidized housing site and set up a mutually agreeable time for recruitment, study staff will be present on site to conduct community engagement "kick-off" events prior to the start of the RCT and distribute flyers at those sites. The goal of these events will be to generate a list of interested participants at each site, and to inform staff and residents at those sites about the frequency with which study staff will be present on site for recruitment and follow-ups. Study staff will also do in-person outreach by going door-to-door to inform residents about the study. We have used these same procedures in our ongoing RCT in permanent supportive housing. We will continue these recruitment procedures until we have reached our target sample size for the RCT. Within each intervention site, we will recruit 2-3 staff to participate in a LHW training. LHW training will be voluntary. LHWs will be recruited via email or direct in-person outreach when study staff are on site.

Retention plan. Given know barriers to participating in research among racial/ethnic minorities, and effective strategies for recruiting and retaining subjects, we developed a **24-point retention plan** to facilitate recruitment/enrollment/retention and acceptance of the study protocols. While our experience supports our estimates of retention rates of 80% at 6 months, we have powered the smoke-free home study using more conservative retention rate of 75% at 6-month follow-up. We have recruited Our 24-point culturally tailored plan includes:

- Trained ***study staff experienced in working with populations that have experienced housing instability or homelessness, who are aware of racial/ethnic disparities, and are familiar with fieldwork locations in subsidized housing***.
- ***Staff training in cultural competency regarding racial/ethnic disparities***, culturally-responsive clinical research practices, and HIPAA.
- Study materials delivered in **participants preferred language** (Chinese, English, Spanish, or Vietnamese)**.**

- We will apply for a ***Certificate of Confidentiality*** from the NIH to protect the privacy of participants against most legal discovery requests and to address patients' concerns with privacy due to immigration status or legal repercussions.
- Offer participants the option of ***meeting at their preferred*** time to complete follow-up assessments (e.g., after work, after school, on the weekends) and at a location of their preference in their community (e.g., local coffee shops, outdoor locations, and community centers).
- Use of ***personalized and culturally sensitive mailings*** such as holiday cards to ensure that patients feel like they are part of our community.
- ***Community Advisory Board*** to include mostly persons with lived experiences of housing instability and living in subsidized housing, housing staff in Contra Costa, Oakland, and San Francisco subsidized housing, community members, key housing policy makers, and local tobacco control experts who will provide guidance on study procedures and assist in the interpretation and dissemination of findings (meeting 2x/year).
- A study design ***grounded in the local culture*** based on housing input, housing needs, and experience with providing services to low-income residents in subsidized housing.
- Conduct ***reminders*** (calls/text-messages/e-mails) one week and 24 hours before the brief intervention visit and research assessments. This is to remind participants with busy schedules.
- Have an ***international toll-free number*** for participants to contact us.
- ***Monetary incentives*** to complete the baseline ($20), and follow-up assessments at 3-months ($15) and 6-months ($25), and an additional $5 for placement of the gas-phase nicotine samplers.
- ***Use of existing procedures to locate patients lost-to-follow up***, including working with housing staff to identify participants so that study staff can meet them at the housing site to conduct or schedule the research assessment or schedule the intervention session; and obtaining the updated contact information for study participants and their designated contact person.
- ***Locator guides*** with contact information for the participants, emergency contacts, friends and medical providers.
- Conduct ***monthly interim re-contact*** calls that are incentivized ($5 visit) for participants at months in between scheduled visits to thank them for participating, remind them of the study incentives, update Locator Guides, and address any questions.
- ***Follow-up and re-contact efforts*** for intervention sessions will include weekly in-person outreach, written letters or e-mail, followed by telephone calls (at least three weekly attempts will be made before shifting to monthly contacts).[52]
- Study instruments and intervention materials designed to the meet the ***literacy needs*** of persons with low educational attainment.

- ***Update participant contact information*** in Locator Guides at follow-up assessments, coaching sessions, and interim re-contact calls.
- Extra effort ***tracking participants lost to follow-up*** at participants' homes, friends' homes, rehabilitation programs, and in outdoor locations where people may spend time, and recording the number of attempts to reach a participant.
- Opportunity to earn a ***$25 bonus*** for participating in all study procedures at 6 months.
- Designated ***tracking/scheduling staff***.
- Use of laminated ***appointment* study business cards** with study contact information.
- Collect ***agency contact information*** from participants where they might receive social services.
- ***Study website*** with educational materials, community resource links, and contact information for study staff.
- Making "***home visits" in subsidized housing and/or outdoor locations*** for difficult-to-find patients.

Reimbursement plan. We will reimburse all resident participants with $20 gift cards for completing the baseline questionnaire, $15 for the 3-month questionnaire and $25 for the 6-month questionnaire. We will reimburse participants $5 for each monthly tracking visit in between follow-ups. Participants selected for random air nicotine monitoring at 6 months will be reimbursed $5 for placing monitors in their home for 7 days. Participants who completed all study procedures will be offered an additional $50 at 6 months follow-up. These amounts have been used in prior studies with minimal risk of coercion.[1,48,53] We will provide lay health workers $20 for completing the baseline questionnaire, $15 for completing the 3-month follow-up questionnaire, and $25 for completing the 6-month follow-up questionnaire.

4.3 Inclusion of Women and Minorities

4.3.1 Eligibility of Women and Minorities

Individuals of any sex/gender, race, or ethnicity may participate.

4.3.2 Recruitment of Women and Minorities

The study recruitment strategy aims to achieve representation of minority groups that reflects the demographics of the affected population in the catchment area. About 30% of subsidized housing residents have limited English proficiency or are monolingual in Chinese, Spanish, or Vietnamese. Estimates of targeted enrollment are based on the demographic distribution of Contra Costa, Oakland, and San Francisco Housing Authority resident population. We will select sites based on the property portfolio of each Housing Authority and identify sites where smoke-free policies don't exist or where some policies exist, but they are inconsistently enforced and residents smoke in their units. Our partners at the Contra Costa, Oakland and San Francisco Housing Authorities provided us demographic estimates of their resident population, which we use to plan our study sample. Of the resident population in San Francisco, 42% are African American, 26% are Asian, 18% are White, 10% are Hispanic/Latino, 1% are American Indian or Alaskan Native, 1% Native Hawaiian or Pacific Islander, and 3% are of mixed race and/or another race/ethnicity, and 50% are female. Of the resident population in Oakland, 54% are African American, 20% Asian, 10% Hispanic/Latinx, 12% White, 1% American Indian or

Alaskan Native, 1% Native Hawaiian or Pacific Islander, 2% mixed race, and 50% female. Of the resident population in Contra Costa, 40% are African American, 12% Asian, 22% are White,19% Hispanic/Latinx, 3% American Indian or Alaskan Native, 2% are Native Hawaiian or Pacific Islander, and 2% are of mixed race and/or other race/ethnicity, and 50% female. We will use our existing data to inform targeted enrollment for transgender participants, estimating that approximately 4% of our sample to identify as transgender.

4.3.3 Age Range of Participants

(A) <u>For studies enrolling only individuals ages 18 and over:</u>

Individuals aged 18 and over are eligible for this study. The proposed study will include all adult participants across the lifespan, without any restrictions based on age, gender or race/ethnicity. Eligible participants will include those who are ≥ 18 years of age. However, no children under the age of 18 years of age will be included in this study of adult cigarette smokers. While federally subsidized housing residents who participate in the smoke-free home intervention might have children living with them in their home, the unit of analysis will be the residents and not their children. However, we will gather information both quantitatively and qualitatively on whether children live in their homes, and the influence, if any, that children had on residents' adoption of a voluntary smoke-free home and the benefit that a smoke-free home might have had in reducing tobacco exposure in the household.

4.3.4 Study Design/Recruitment Considerations Related to Age Groups

The study design and recruitment strategy aim to achieve representation of age groups that reflect the demographics of the affected population – i.e., adults aged 18 years or older who self-identify as racial/ethnic minority, and who may speak English, Chinese, Spanish, or Vietnamese.

4.4 Participant Registration

A written, signed, informed consent form (ICF) and a Health Insurance Portability and Accountability Act (HIPAA) authorization must be obtained before any study-specific assessments are initiated. A copy of the signed ICF will be given to the subject and a copy will be filed in the medical record. The original will be kept on file with the study records.

All participants consented to the study will be registered in OnCore®, the UCSF Helen Diller Family Comprehensive Cancer Center Clinical Trial Management System (CTMS). The system is password protected and meets HIPAA requirements.

4.5 Randomization/Assignment to Intervention

Randomization and allocation. We will randomize 24 subsidized housing sites into intervention and waitlist control arms. We will use the method of restricted randomization—commonly applied in cluster-randomized trials with small numbers of clusters—to ensure an acceptable level of balance across groups at baseline.[54] First, we will create two sets of 12 housing sites balanced on preexisting variables describing relevant characteristics of sites and their residents (e.g., property size, smoking-related policies, and geography). Second, we will randomly assign one set of sites to intervention and the other to wait-list control. If we are not meeting our sample size targets with the 24 housing sites, we will randomize additional sites into intervention and control groups.

4.6 Blinding

Not applicable

5 Study Intervention

5.1 Administration and/or Delivery of Study Intervention

Smoke-free home resident intervention. The study's investigators will train bilingual study staff to deliver the intervention to residents using a script that matches the content in the smoke-free home intervention pamphlet. The in-person delivery of the intervention and pamphlet will be the primary modes of intervention delivery to residents. The pamphlet will include: (1) the harms of tobacco, e-cigarette use, cannabis use and exposure (secondhand and thirdhand), (2) an exercise to calculate personal cost of tobacco use, (3) benefits of a smoke-free home, (4) skill-building on how to adopt a smoke-free home, and (5) motivational language on smoke-free home adoption. The study staff will qualitatively assess participants' knowledge by prompting questions on the topics covered and will refer participants to LHWs for one-on-one coaching. Participants will receive a pledge to designate their homes smoke-free.

LHW coaching. LHWs will deliver brief tobacco cessation coaching by engaging with residents within 2 weeks of the smoke-free home resident intervention, and on a monthly schedule as part of their routine encounters with residents (total 6 sessions). The goal of the LHW coaching is to provide support tailored to residents' readiness for making changes towards smoke-free home adoption and/or tobacco cessation. During the brief coaching session (10 to 15 minutes), LHWs will use an action form to engage residents by first assessing their readiness for smoke-free home adoption and/or tobacco cessation, and to engage residents in committing to at least one action item that matches the resident's readiness for change. At each encounter, LHWs will complete an assessment form that includes attempts to reach participants, an assessment of their readiness to quit, and an action plan. The action plan will include prompts to: 1) talk to family or friends, 2) make changes towards smoke-free home adoption and/or tobacco cessation, 3) call Kick It California or Asian Smokers' telephone Quitline, [55,56] and 4) talk to their healthcare provider or neighborhood pharmacy (if they do not have a healthcare provider) for cessation counseling. Study staff will hold monthly Lay Health Worker Learning Collaboratives for supervision and to mutually problem solve questions. *Fidelity assessment*. Because resident-LHW encounters will take place as part of routine care, study staff will request permission to conduct in-person observations of a random sample of resident-LHW dyads (~10% of these interactions). As described above, we will use a checklist to determine whether LHWs delivered intervention content and provide feedback on observed departures.

5.2 Interventionist Training and Tracking

LHW training. The study's investigators will train study staff to deliver a 1-hour LHW training to housing staff in a group setting using an interactive webinar with didactics, team-building and role-playing activities using our previously-developed training materials for LHWs. [41-43,57-59] The training will include: a) education on tobacco, e-cigarettes and cannabis use and exposure (secondhand and thirdhand smoke),[55,56] 2) talking points on engagement, 3) skill building using a flip chart to provide smoking cessation coaching using Ask, Advise and Refer,[60] and 4) setting individual action plans on tobacco cessation. We will provide LHWs a laminated flip chart that contains a list of talking points in Chinese, English, Spanish, or Vietnamese on one side, and on

the other side a headline, a brief explanatory text, and culturally appropriate graphics for the resident.[41] *Fidelity assessment.* The study's investigators will observe study staff delivering the LHW training (~10% of trainings). We will keep a checklist of topics covered, the time spent on each topic, and assess whether there were departures in delivery by language.[61] Departures from the protocol will be discussed to encourage better adherence.

Study staff training to deliver resident intervention: Study staff will receive training on nicotine addiction; tobacco, e-cigarette, and cannabis co-use; harms related to tobacco exposure (secondhand and thirdhand); and treatment for tobacco cessation. Study staff will receive training in motivational interviewing [62] that will reinforce residents' sense of autonomy and self-efficacy as they take steps toward behavior change. Study staff will receive a laminated card that includes talking points in Chinese, English, Spanish, or Vietnamese for each page of the pamphlet that they will role-play in the training. After learning the intervention content, study staff will have a chance to deliver the intervention to the study team and receive feedback on delivery. They will be observed in the field delivering the intervention to resident participants and receive feedback. *Fidelity assessment.* The PI and bilingual co-investigators will conduct in-person observations of study staff delivering the resident intervention and lay health worker training (~10% of resident and lay health worker training). We will use a previously-developed check-list to assess whether study staff and lay health workers delivered the intervention content, engaged with residents, presented materials correctly (e.g., adherence to flip chart instructions), and developed an action plan.[41]

For all observations for LHW and study staff delivering the resident intervention, we will keep a checklist of topics covered, the time spent on each topic, and assess whether there were departures in delivery by language.[61] Observed departures from the protocol will be discussed with study staff to encourage better adherence.

5.3 Modifications to Administration of the Intervention and/or Supportive Care

Not applicable

5.4 Adherence Assessment

We will assess adherence to the LHW coaching sessions.

5.5 Concomitant Therapy

Not applicable

5.5.1 Allowed Therapy

Not applicable

5.5.2 Required Therapy

Not applicable

5.5.3 Prohibited Therapy

Not applicable

5.6 Participant Discontinuation/Withdrawal from the Study

Participants are free to withdraw from participation in the study at any time upon request.

An investigator may discontinue a participant from the study for the following reasons:

- Unacceptable adverse event(s)
- Significant study intervention non-compliance, unless varying compliance is an aspect of the study objectives
- Lost-to-follow up; unable to contact participant (see Section 5.7 - Lost to Follow-Up)
- Any event or medical condition or situation occurs such that continued collection of follow-up study data would not be in the best interest of the participant or might require an additional treatment that would confound the interpretation of the study
- The participant meets an exclusion criterion (either newly developed or not previously recognized) that precludes further study participation

5.7 Lost to Follow-up

A participant will be considered lost to follow-up if he or she fails to return for any scheduled visits after their last visit in the study, and study staff are unable to contact the participant after at least 3 attempts. Before a participant is deemed lost to follow-up, the investigator or designee will make every effort to regain contact with the participant (where possible, 3 telephone calls and/or in person visits and, if necessary, a letter to the participant's last known mailing address or local equivalent methods). These contact attempts will be documented in the participant's study file. Should the participant continue to be unreachable, he or she will be considered to have withdrawn from the study with a primary reason of lost to follow-up.

We will employ techniques for tracking participants that we have used in past studies to maintain high rates of follow-up: these include monthly face-to-face or telephone check-in visits and locating participants lost to follow-up.[1,45-48,53,63-65]Participants will be incentivized $5 for each monthly check-in visit, in between study visits. At enrollment, we will ask participants to provide as many forms of contact information as possible including names of family/friends and case managers, telephone numbers, addresses of places where they usually stay or can be found, and to update this information monthly. At the enrollment visit, study staff will request consent to contact participants to remind them about upcoming study visits via cellphone. If participants miss any visit (scheduled study visit or check-in) and the participant has consented to it, study staff will conduct participant tracking. Tracking involves study staff calling people whose name and contact information the participant has given the study staff for this purpose ("contacts"). Staff, when calling on these contacts, will request help in locating the participant and/or will leave a message for the participant. Study staff will tell contacts only that the participant is enrolled in a research study. If the participant has given permission to do so, the study staff may go to look for the participant at a place where the participant has said that he/she frequents. When doing this, the staff may bring along a picture of the participant to assist in locating the participant. With these follow-up procedures, we have achieved rates of retention >85% for longitudinal studies with homeless and PSH participants.[45-48,64,65] In our pilot smoke-free home intervention study, we achieved retention rates of 86% and 84% at 3- and 6-months follow-up.[1] In our ongoing clinical trial in PSH, retention rates at 3-months is 93% and 88% at 6-months.

6 Study Procedures and Assessments

6.1 Schedule of Activities

Note: If the study involves multiple cohorts/arms, consider creating a schedule for each cohort/arm in order to more clearly identify cohort/arm-specific activities.

**Assessments/Procedures**	**Screening**	**Study Intervention Period**					**End of Study Intervention**	**Follow-up**
**Study Visit / Day (Window, # Days)**	**Visit -1 /Day 0**	**Visit 1 /Day 1 (7 days)**	**Visit 2 / D 90 (30 days)**	**Visit 3 / D 180 (30 days)**	**Visit 4 / D 270 (30 days)**	**Visit 5 / D 360 (30 days)**	**Visit 6 / D 360 (30 days)**	**Every 3 months**
Informed Consent[1]	X							
Inclusion/Exclusion Criteria	X							
Concomitant Medications	X	X	X	X	X	X	X	
Adverse Events	X	X	X	X	X	X	X	X
Randomization/Assignment to Intervention	X							
Administration of Study Intervention to intervention group		X	X	X	X	X		
Protocol-Specific Assessments/Procedures [2]	X	X	X	X	X	X	X	X
Administration of study intervention to wait-list control group				X	X	X	X	

[2] Check-in visits in between scheduled visits at 3 months and 6 months follow-up can take place by telephone.

[1] Informed consent must be obtained prior to any study-specific procedures and may be obtained prior to the screening window.

6.2 Study Procedures and Assessments

6.2.1 Screening Period / Visit -1 (Day 0 to Day 1)

After an individual provides informed consent, the following activities will be performed during the Screening Period:

- Inclusion/exclusion criteria review
- Adverse Events assessment
- Protocol-specific assessments/procedures
- Randomization/Assignment to Intervention

6.2.2 Study Intervention Period

6.2.2.1 Visit 1 (Day 1 +/- 7 days)

- Adverse Events assessment
- Protocol-specific assessments/procedures
- Administration of Study Intervention

6.2.2.2 Visit 2 (Day 90 +/- 30 days)

- Adverse Events assessment
- Protocol-specific assessments/procedures

6.2.2.3 Visit 3 (Day 180 +/- 30 days)

- Adverse Events assessment
- Administration of Study Intervention to wait-list control group
- Protocol-specific assessments/procedures
- Placement of air nicotine samplers

6.2.2.4 Visit 4 (Day 270 +/- 30 days)

- Adverse Events assessment
- Protocol-specific assessments/procedures

6.2.2.5 Visit 5 (Day 360 +/- 30 days)

- Adverse Events assessment
- Protocol-specific assessments/procedures

6.2.3 End of Study Intervention / Visit 6 (Day 360 +/- 30 days)

- Adverse Events assessment

- Protocol-specific assessments/procedures
- Exit interviews

6.2.4 Follow-up

Participants will be followed every 3 months for up to 6 months after discontinuing the study intervention. The following procedures will be performed at each follow-up time point:

- Adverse Events assessment
- Protocol-specific assessments/procedures

7 Reporting and Documentation of Results

7.1 Measures and Instruments

Data collection. Questionnaires designed in REDCap will be administered by study staff using an iPad. Intervention and wait-list control participants will complete questionnaires at baseline, 3-, and 6 months follow-up. Wait-list control group participants who cross over to the intervention arm will also complete questionnaires at 9- and 12-months follow-up (3- and 6-months following the intervention).

Resident measures (Table 2). Demographics. We will obtain information on age, sex (sex assigned at birth, current gender), race/ethnicity, education, income from all sources, length of stay in residence for all household members, marital status, number of smokers and nonsmokers in the household, the age and gender of each household member, and whether any non-smoking household member is pregnant.

Social determinants of health. We will obtain information on unmet needs,[66] social and instrumental support,[67,68] residential history, [53,69,70] homelessness history,[53,69,70] and exposure to urban life stressors.[71]

Neighborhood-level factors. We will ask where participants obtained their tobacco products, the distance they had to travel to obtain those products, how far their housing was from tobacco, alcohol, and cannabis retailers, and perceptions of ease with which they could obtain such products.[1]

Alternative tobacco product and cannabis use. We will assess ever and past 30-days use and indoor use of e-cigarettes, cigars/little cigars, smokeless tobacco, hookah, blunts, and cannabis.

Tobacco dependence and smoking cessation. We will use the Fagerstrom's test for nicotine dependence,[72] assess dependence for alternative tobacco products,[72,73] craving [74], impulsivity [75], quit attempts (ever, in the past year, and since the last visit), length of the last quit attempt, and use of cessation aids.[76]

Smoke-free policy measures.[1,34] We will assess knowledge of current no-smoking policies, frequency of past-month exposure to tobacco, e-cigarettes and cannabis, and attitudes toward smoke-free policies and indoor use of e-cigarettes and cannabis.[34]

Measures related to voluntary smoke-free home adoption.[1,34] We will ask whether participants adopted a smoke-free home voluntarily in the past 90 days and the length of the

last adoption, which will be used to define our primary outcome. Those who report a smoke-free home will be asked when they last smoked in their home. Among those who report using cannabis indoors, we will explore whether participants voluntarily adopted a cannabis-free home and the length of time they were cannabis free.

Expenditures for tobacco use. We will obtain information on the amount of money spent on tobacco in the past week, which we will use to estimate tobacco-related expenditures.[1]

Chronic diseases. Using the National Health Interview Survey, we will ask about diagnoses of liver disease, renal disease, heart disease, hypertension, diabetes, cancer, pulmonary disease, and HIV.[77]

COVID-19 response. We will ask participants to describe: (1) ways in which COVID-19 has impacted their tobacco use and cessation behaviors,[78] (2) whether they were diagnosed and/or exposed to someone with COVID-19, (3) whether they are able to socially distance and wear masks, (4) their tobacco use and COVID-19 harm perceptions,[79] and (6) how COVID-19 has influenced their engagement with the intervention.

Mental health. We will screen for depression using the 10-item Center for Epidemiologic Studies Depression Scale, [80] anxiety using a seven-item anxiety scale (Generalized Anxiety Disorder-7),[81] and post-traumatic stress disorder using the Primary Care PTSD Screen.[82]

Alcohol and substance use disorders. We will administer the Alcohol, Smoking and Substance Involvement Screening Test version 3.0 (WHO-ASSIST).[83-86] To assess volume consumed, we will administer the Alcohol Use Disorders Identification Test (AUDIT-C).[87,88]

Satisfaction and usefulness of the intervention. At 3- and 6-months follow-up, we will evaluate satisfaction using a Likert scale and usefulness using a previously validated item by asking, 'In the past three months, what role did the intervention play in helping you adopt a smoke-free home or quit smoking?'

Exposure to lay health worker coaching. At 3- and 6-months follow-up, we will ask residents whether they had monthly coaching encounters with lay health workers and the number of these encounters, whether they were asked and advised to quit by lay health workers, and satisfaction and usefulness of these encounters.

Site-level changes to smoke-free policies. At 3- and 6-months follow-up, we will ask participants who received the intervention whether they had discussed the intervention or shared materials with family, neighbors and/or staff in their building, the number of people with whom they had discussed the intervention, and whether there were site-level changes to smoke-free policies (e.g., new rules around smoking indoors).

Lay health worker measures. LHWs will be asked to complete the Smoking Knowledge Attitudes Practices (SKAP) survey at baseline, 3- and 6-month follow-ups.[89,90] The SKAP survey assesses knowledge, attitudes, barriers, efficacy and practices related to providing tobacco treatment.[89] We will ask about demographics, job roles, smoking history (if applicable), policies around smoking (e.g., policies restricting tobacco, e-cigarette and/or cannabis use indoors, provision of on-site cessation services), enforcement of smoke-free policies (complaints, warnings, or evictions), and barriers to enforcement of current policies. LHWs will complete a

monthly assessment form that documents attempts to reach participants, an assessment of participants' readiness to quit, and an action plan.

8 Adverse Events and Serious Adverse Events

8.1 Definition of Adverse Event

An adverse event (AE) is defined as any untoward medical occurrence associated with the use of an intervention in humans, whether or not considered intervention related.

8.2 Definition of Serious Adverse Event

An AE that results in any of the following outcomes is defined as a Serious Adverse Event:

- Death,
- Life-threatening adverse experience*,
- Inpatient hospitalization or prolongation of existing hospitalization,
- Persistent or significant disability/incapacity,
- Congenital anomaly/birth defect, or cancer, or
- Any other experience that suggests a significant hazard, contraindication, side effect or precaution that may require medical or surgical intervention to prevent one of the outcomes listed above,
- Event that changes the risk/benefit ratio of the study.

*A life-threatening adverse experience is any AE that places the patient or subject, in the view of the investigator, at immediate risk of death from the reaction as it occurred, i.e., it does not include a reaction that, had it occurred in a more severe form, might have caused death.

8.3 Classification of Adverse Events

8.3.1 Severity

Adverse events are graded according to the Common Terminology Criteria for Adverse Events (CTCAE) as developed and revised by the Common Therapy Evaluation Program (CTEP) of the National Cancer Institute.

8.3.2 Attribution

Adverse events are further given an assignment of attribution or relationship to study intervention or procedure. Attribution categories are:

- **Definite** – The adverse event is clearly related to the study intervention or procedure.
- **Probable** – The adverse event is likely related to the study intervention or procedure.
- **Possible** – The adverse event may be related to the study intervention or procedure.
- **Unrelated** – the adverse event is clearly not related to the study intervention or procedure.

8.3.3 Expectedness

An adverse event is considered unexpected if it is not listed in the investigator brochure or package insert(s), or is not listed at the specificity or severity that has been observed, or, if an investigator brochure is not required or available, the event is not consistent with the risk information described in the general investigational plan or elsewhere in the current application.

8.4 Adverse Events Monitoring

This study is a minimal risk level study that does not require monitoring by the HDFCCC Data and Safety Monitoring Committee (DSMC) as per the National Cancer Institute-approved Data and Safety Monitoring Plan. Ultimately, the PI is responsible for the safety and conduct of this study.

8.5 Follow up of Adverse Events

All participants who experience adverse events will be followed with appropriate medical management until resolved or stabilized, as determined by the investigator.

8.6 Documenting and Reporting of Adverse Events

Adverse Events will be documented in the study Case Report Forms (CRFs) and reported to the IRB, HDFCCC DSMC, and collaborators in accordance with all applicable institutional and regulatory requirements.

9 Statistical Considerations

9.1 Sample Size Considerations

9.1.1 Sample Size and Power Estimate

The sample size calculation was based on the primary outcome of smoke-free home adoption ≥90 days at 6-months follow-up. We also conducted power calculations for the secondary outcome of PPA at 6-months follow-up. Power analyses assumed 80% power; intra-cluster correlation of 0.01, two-tailed alpha=0.05; and 75% retention at 6 months follow-up. With these parameters, we will need N=544 residents who smoke, recruited from 24 housing sites to detect a difference of 11.5% for the primary outcome of smoke-free home adoption, with 12% for the control arm versus 23.5% for the intervention arm (OR=2.25), which compares favorably with 31.3% obtained in the pilot study.[1] For the secondary outcome of PPA at 6 months, the sample size provides 86.6% power to detect a difference of 11% (13% versus 4%), which compares favorably with 16.9% obtained in the pilot.[1]

9.1.2 Randomization and Blinding

We will randomize 24 subsidized housing sites into intervention and waitlist control arms (Figure 5). We will use the method of restricted randomization—commonly applied in cluster-randomized trials with small numbers of clusters—to ensure an acceptable level of balance across groups at baseline.[54] First, we will create two sets of 12 housing sites balanced on preexisting variables describing relevant characteristics of sites and their residents (e.g., property size, smoking-related policies, and geography). Second, we will randomly assign one set of sites to intervention and the other to wait-list control.

9.1.3 Stratification Factors

We will stratify by current smokefree policies, geography, and property size.

9.1.4 Accrual Estimates

Our total sample size is 544 resident participants, and we expect to complete recruitment within 24 to 30 months.

9.2 Interim Analyses and Stopping Rules

Not applicable

9.3 Statistical Analysis Plans

Descriptive analyses. We will estimate means and proportions, variation, and confidence intervals for demographic and other covariates. We will consider relevant biological variables such as age, sex, and race/ethnicity, and assess whether they are balanced at baseline by randomization (randomization check) and attrition (attrition analysis). Multiple imputation will allow use of all available data while assuming that data are missing at random, conditional on modeled variables.[91-93]

9.3.1 Analysis Populations

Not applicable

9.3.2 Primary Analysis (or Analysis of Primary Endpoints)

Primary analyses include intention-to-treat comparisons. The 3-level data structure—including subsidized housing site, residents, and repeated assessments—will be accommodated by logistic mixed effects models with random intercepts for sites and residents. The primary outcome will be a binary indicator of adopting a smoke-free home which, in an intention-to-treat analysis, will be regressed onto indicators of experimental groups, categorical time (baseline, 3 months, 6 months), and the group-by-time interaction. Any significant intervention group main effects at follow-up and group-by-time interaction effect will be interpreted and described. If any key demographic or risk factors are imbalanced at baseline, we will include a propensity score-based adjustment.[94-96]

9.3.3 Secondary Analysis (or Analysis of Secondary Endpoints)

We will conduct several secondary analyses to supplement the primary analyses on the primary outcome and to examine the effects on secondary outcomes. First, we will fit a mixed effects logistic model of the secondary outcome, point prevalence abstinence. Second, participants lost-to-follow-up will be compared to those who completed the study to assess whether there are systematic differences in baseline covariates between the two groups. We will conduct sensitivity analyses to assess whether the results of the outcomes analyses will change if the mixed effects models included baseline covariates that differed between participants lost-to-follow and those who completed the study. Third, we will include sex, race/ethnicity, and their interactions with the group and time in the logistic mixed effects model to examine if there is any sex and/or race/ethnicity differences in the intervention effects on the outcomes. Fourth, we will perform mediation analyses to identify the mechanisms/pathways by which the intervention may

have impacted smoke-free home adoption or PPA outcomes including change in smoke-free policy attitudes, exposure to lay health workers, neighborhood environment, and fidelity of intervention delivery. Fifth, we will explore moderation effects of children or pets at home, indoor cannabis use or other combustible tobacco use on intervention outcomes of smoke-free homes and PPA outcomes via mixed effects regression models. Replicability of within-group changes on primary and secondary outcomes will be tested by comparing baseline to 6-month changes in the intervention group to corresponding 6- to 12-month changes in the waitlist group.

9.3.4 Exploratory/Correlative Analysis/Assessments

Analyses of lay health worker data will include a pre-post training analysis of the smoking knowledge, attitudes, practices, efficacy, and barriers scales using mixed linear models. We will fit mixed linear regression models to examine factors associated with change in each of the scales, clustering by site and participant ID, and adjusting for demographics, geographic location, lay health worker smoking status, coaching encounters with residents, and lay health worker role.

10 Study Management

10.1 Pre-study Documentation

Before initiating this trial, the PI will have written and dated approval from the Institutional Review Board for the protocol, written informed consent form, subject recruitment materials, and any other written information to be provided to participants before any protocol related procedures are performed on any participants.

The PI must comply with GCP/ICH guidelines and all applicable regulatory requirements.

10.2 Institutional Review Board Approval

The protocol, the proposed informed consent form, and all forms of participant-facing materials related to the study (e.g., advertisements used to recruit participants) will be reviewed and approved by the IRB. The initial protocol and all protocol amendments must be approved by the IRB prior to implementation.

10.3 Informed Consent

All participants must be provided a consent form describing the study with sufficient information for each participant to make an informed decision regarding their participation. Participants must sign the IRB-approved informed consent form prior to participation in any study specific procedure. The participant must receive a copy of the signed and dated consent document. The original signed copy of the consent document must be retained in the medical record or research file.

10.4 Changes in the Protocol

Once the protocol has been approved by the IRB, any changes to the protocol must be documented in the form of an amendment. The amendment must be signed by the PI and approved by the IRB prior to implementation.

If it becomes necessary to alter the protocol to eliminate an immediate hazard to participants, an amendment may be implemented prior to IRB approval. In this circumstance, however, the PI must then notify the IRB according to institutional requirements.

10.5 Case Report Forms (CRFs)

The PI and/or designee will prepare and maintain adequate and accurate participant case histories with observations and data pertinent to the study. Study specific Case Report Forms (CRFs) will document study data for safety monitoring and data analysis. All study data will be entered into OnCore® or other CTMS used for the study via standardized CRFs in accordance with the CTMS study calendar, using single data entry with a secure access account. Study personnel will complete the CRFs; the PI will review and approve the completed CRFs.

The information collected on CRFs shall be identical to that appearing in original source documents. Source documents will be found in the participant's medical records maintained by study personnel. All source documentation should be kept in separate research files for each participant.

In accordance with federal regulations, the PI is responsible for the accuracy and authenticity of data entered onto CRFs. The PI will approve all completed CRFs to attest that the information contained on the CRFs is true and accurate.

All source documentation and CTMS data will be available for review/monitoring.

10.6 Record Retention

The PI is required to prepare and maintain adequate and accurate case histories that record all observations and other data pertinent to the investigation on each study participant. Study documentation includes all CRFs, data correction forms or queries, source documents, Sponsor-Investigator correspondence, monitoring logs/letters, and regulatory documents (e.g., protocol and amendments, IRB correspondence and approval, signed participant consent forms). Source documents include all recordings of observations or notations of clinical activities and all reports and records necessary for the evaluation and reconstruction of the clinical research study. The PI shall retain records for a period of 2 years following the conclusion of the study.

10.7 Publications

The preparation and submittal for publication of manuscripts containing the study results shall be in accordance with a process determined by mutual written agreement among the Sponsor-Investigator and collaborators.

11 References

1. Durazo A, Hartman-Filson M, Perez K, Alizaga NM, Petersen AB, Vijayaraghavan M. Smoke-Free Home Intervention in Permanent Supportive Housing: A Multi-Faceted Intervention Pilot. *Nicotine Tob Res*. Mar 3 2020;doi:10.1093/ntr/ntaa043
2. Martell BN, Garrett BE, Caraballo RS. Disparities in Adult Cigarette Smoking - United States, 2002-2005 and 2010-2013. *MMWR Morb Mortal Wkly Rep*. Aug 5 2016;65(30):753-8. doi:10.15585/mmwr.mm6530a1
3. Guan A, Kim-Mozeleski JE, Tan JY, et al. Serious quit attempts and cessation implications for Asian American male smokers. *Addict Behav*. Jan 2020;100:106129. doi:10.1016/j.addbeh.2019.106129
4. Chang E, Dove M, Saw A, Tsoh JY, Fung LC, Tong EK. Home Smoking Bans and Urinary NNAL Levels to Measure Tobacco Smoke Exposure in Chinese American Household Pairs. *Int J Environ Res Public Health*. Jul 20 2021;18(14)doi:10.3390/ijerph18147682
5. U.S. Department of Health and Human Services. Atlanta: U.S. Department of Health and Human Services CfDCaP, National Center for Chronic Disease Prevention and Health Promotion, Office on Smoking and Health, 1998 [accessed 2018 Jun 6]. Tobacco Use Among U.S. Racial/Ethnic Minority Groups—African Americans, American Indians and Alaska Natives, Asian Americans and Pacific Islanders, Hispanics: A Report of the Surgeon General. . *Atlanta: US Department of Health and Human Services, Centers for Disease Control and Prevention, National Center for Chronic Disease Prevention and Health Promotion, Office on Smoking and Health, 1998* Available at: https://www.cdc.gov/tobacco/data_statistics/sgr/1998/index.htm; Accessed February 21, 2022
6. Tsai J HD, Gentzke A.S. et al. Exposure to Secondhand Smoke Among Nonsmokers — United States, 1988-2014. *MMWR - Morbidity and Mortality Reports*. 2018;67(48):134-1346
7. Hiatt B. The Impact of Cancer in San Francisco -- San Francisco Cancer Initiative. *http://wwwsfcancerorg/*. 2016;
8. NCI 2021. Seer Cancer Statistics Review -TaSEAF, 2022].
9. Trinidad DR, Xie B, Fagan P, et al. Disparities in the Population Distribution of African American and Non-Hispanic White Smokers Along the Quitting Continuum. *Health Educ Behav*. Mar 20 2015;doi:10.1177/1090198115577376
10. Trinidad DR, Perez-Stable EJ, Emery SL, White MM, Grana RA, Messer KS. Intermittent and light daily smoking across racial/ethnic groups in the United States. *Nicotine Tob Res*. Feb 2009;11(2):203-10. doi:ntn018 [pii]
10.1093/ntr/ntn018
11. Trinidad DR, Perez-Stable EJ, White MM, Emery SL, Messer K. A nationwide analysis of US racial/ethnic disparities in smoking behaviors, smoking cessation, and cessation-related factors. *Am J Public Health*. Apr 101(4):699-706. doi:AJPH.2010.191668 [pii]
10.2105/AJPH.2010.191668
12. Max W, Sung HY, Tucker LY, Stark B. The disproportionate cost of smoking for African Americans in California. *Am J Public Health*. Jan 2010;100(1):152-8. doi:10.2105/AJPH.2008.149542
13. Max W, Sung HY, Shi Y. The cost of secondhand smoke exposure at home in California. *Tob Control*. Mar 2015;24(2):205-10. doi:10.1136/tobaccocontrol-2013-051253
14. Miller KD, Ortiz AP, Pinheiro PS, et al. Cancer statistics for the US Hispanic/Latino population, 2021. *CA Cancer J Clin*. Nov 2021;71(6):466-487. doi:10.3322/caac.21695
15. Dominguez K, Penman-Aguilar A, Chang MH, et al. Vital signs: leading causes of death, prevalence of diseases and risk factors, and use of health services among Hispanics in the United States - 2009-2013. *MMWR Morb Mortal Wkly Rep*. May 8 2015;64(17):469-78.

16. Sebrie EM, Sandoya E, Bianco E, Hyland A, Cummings KM, Glantz SA. Hospital admissions for acute myocardial infarction before and after implementation of a comprehensive smoke-free policy in Uruguay: experience through 2010. *Tob Control*. Nov 2014;23(6):471-2. doi:10.1136/tobaccocontrol-2012-050954
17. Lightwood JM, Glantz SA. Declines in acute myocardial infarction after smoke-free laws and individual risk attributable to secondhand smoke. *Circulation*. Oct 6 2009;120(14):1373-9. doi:CIRCULATIONAHA.109.870691 [pii]10.1161/CIRCULATIONAHA.109.870691
18. Hahn EJ, Rayens MK, Wiggins AT, Gan W, Brown HM, Mullett TW. Lung cancer incidence and the strength of municipal smoke-free ordinances. *Cancer*. Jan 15 2018;124(2):374-380. doi:10.1002/cncr.31142
19. Instituting Smoke-Free Public Housing. 24 CFR Parts 965 and 966 Department of Housing and Urban Development; 2016. p. 15.
20. Thorpe LE, Feinberg AM, Elbel B, et al. Time to Track Health Outcomes of Smoke-Free Multiunit Housing. *Am J Prev Med*. Feb 2018;54(2):320-322. doi:10.1016/j.amepre.2017.09.016
21. Jiang N, Gill E, Thorpe LE, et al. Implementing the Federal Smoke-Free Public Housing Policy in New York City: Understanding Challenges and Opportunities for Improving Policy Impact. *Int J Environ Res Public Health*. Nov 29 2021;18(23)doi:10.3390/ijerph182312565
22. Thorpe LE, Anastasiou E, Wyka K, et al. Evaluation of Secondhand Smoke Exposure in New York City Public Housing After Implementation of the 2018 Federal Smoke-Free Housing Policy. *JAMA Netw Open*. Nov 2 2020;3(11):e2024385.doi:10.1001/jamanetworkopen.2020.24385
23. Vijayaraghavan M, King BA. Advancing Housing and Health: Promoting Smoking Cessation in Permanent Supportive Housing. *Public Health Rep*. Apr 30 2020:33354920922374. doi:10.1177/0033354920922374
24. Sangmo L, Braune T, Liu B, et al. Secondhand marijuana exposure in a convenience sample of young children in New York City. *Pediatr Res*. Mar 2021;89(4):905-910. doi:10.1038/s41390-020-0958-7
25. Zajac L, Gallate X, Gu G, et al. Disparities in Marijuana and Tobacco Smoke Incursions Among New York City Families During Early Months of the COVID-19 Pandemic. *J Public Health Manag Pract*. May-Jun 01 2022;28(3):248-257. doi:10.1097/PHH.0000000000001440
26. Delgado-Rendon A, Cruz TB, Soto D, Baezconde-Garbanati L, Unger JB. Second and Thirdhand Smoke Exposure, Attitudes and Protective Practices: Results from a Survey of Hispanic Residents in Multi-unit Housing. *J Immigr Minor Health*. Oct 2017;19(5):1148-1155. doi:10.1007/s10903-016-0540-x
27. Delgado Rendon A, Cruz TB, Baezconde-Garbanati L, Soto C, Unger JB. Managers' Practices of Tobacco and Marijuana Smoking Policies in Hispanic-Occupied Multiunit Housing. *Health Equity*. 2019;3(1):304-311. doi:10.1089/heq.2018.0100
28. Anastasiou E, Chennareddy S, Wyka K, Shelley D, Thorpe LE. Self-reported Secondhand Marijuana Smoke (SHMS) Exposure in Two New York City (NYC) Subsidized Housing Settings, 2018: NYC Housing Authority and Lower-Income Private Sector Buildings. *J Community Health*. Jun 2020;45(3):635-639. doi:10.1007/s10900-019-00783-x
29. Vijayaraghavan M, Benmarnhia T, Pierce JP, et al. Income disparities in smoking cessation and the diffusion of smoke-free homes among U.S. smokers: Results from two longitudinal surveys. *PLoS One*. 2018;13(7):e0201467. doi:10.1371/journal.pone.0201467
30. King BA, Dube SR, Homa DM. Smoke-free rules and secondhand smoke exposure in homes and vehicles among US adults, 2009-2010. *Prev Chronic Dis*. 2013;10:E79. doi:10.5888/pcd10.120218
31. Mills AL, Messer K, Gilpin EA, Pierce JP. The effect of smoke-free homes on adult smoking behavior: a review. *Nicotine Tob Res*. Oct 2009;11(10):1131-41. doi:ntp122 [pii] 10.1093/ntr/ntp122

32. Vijayaraghavan M, Messer K, White MM, Pierce JP. The effectiveness of cigarette price and smoke-free homes on low-income smokers in the United States. *Am J Public Health*. Dec 103(12):2276-83. doi:10.2105/AJPH.2013.301300
33. Office of Policy Development and Research. Smoke-free Public Housing: Research and Implementation. https://wwwhudusergov/portal/periodicals/em/winter16/highlight2html. 2016.
34. Alizaga NM, Nguyen T, Petersen AB, Elser H, Vijayaraghavan M. Developing Tobacco Control Interventions in Permanent Supportive Housing for Formerly Homeless Adults. *Health Promot Pract*. Apr 11 2019:1524839919839358. doi:10.1177/1524839919839358
35. Petersen AB, Elser H, Nguyen T, Alizaga NM, Vijayaraghavan M. Smoke-Free or Not: Attitudes Toward Indoor Smoke-Free Policies Among Permanent Supportive Housing Residents. *Am J Health Promot*. Jan 2020;34(1):32-41. doi:10.1177/0890117119876763
36. Vijayaraghavan M KM, Handley M, Max W. A smoke-free home intervention in permanent supportive housing. *Available at: https://clinicaltrialsgov/ct2/show/NCT04855357; Accessed on February 12, 2022*. 2022;
37. Kegler MC, Bundy L, Haardorfer R, et al. A minimal intervention to promote smoke-free homes among 2-1-1 callers: a randomized controlled trial. *Am J Public Health*. Mar 2015;105(3):530-7. doi:10.2105/AJPH.2014.302260
38. Michie S M VSM, West R. The behavior change wheel: A new method for characterising and designing behavior change interventions. *Implement Sci*. 2011;6(42):1-11.
39. Mangurian C NG, Schillinger D, Newcomer JW, Dilley J, Handley MA. Utilization of the behavior change wheel framework to develop a model to improve cardiometabolic screening for people with severe mental illness. *Implement Sci*. 2017;12(1):134.
40. Handley MA HE, Gonzalez-Mendez E, Stotland NE et al. . Applying the COM-B model to creation of an IT-enabled health coaching and resource linkage program for low-income Latina moms with recent gestational diabetes: the STAR MAMA program. *Implement Sci*. 2016;11(1):73.
41. Tsoh JY, Burke NJ, Gildengorin G, et al. A Social Network Family-Focused Intervention to Promote Smoking Cessation in Chinese and Vietnamese American Male Smokers: A Feasibility Study. *Nicotine Tob Res*. Aug 2015;17(8):1029-38. doi:10.1093/ntr/ntv088
42. Tsoh J, Burke N, Gildengorin G, et al. Promoting Smoking Cessation among Vietnamese Americans using a Family-Based Lay Health Worker Intervention: A Cluster Randomized Controlled Trial. presented at: Paper accepted for oral presentation at the Society for Research on Nicotine and Tobacco 23rd Annual Meeting; 2017; Florence, Italy.
43. Burke NJ, Phung K, Yu F, et al. Unpacking the 'black box' of lay health worker processes in a US-based intervention. *Health Promot Int*. Dec 1 2018;doi:10.1093/yel/day094
44. Kenny JD, Tsoh JY, Nguyen BH, Le K, Burke NJ. Keeping Each Other Accountable: Social Strategies for Smoking Cessation and Healthy Living in Vietnamese American Men. *Fam Community Health*. Jul-Sep 01 2021;44(3):215-224. doi:10.1097/FCH.0000000000000270
45. Kushel MB, Colfax G, Ragland K, Heineman A, Palacio H, Bangsberg DR. Case management is associated with improved antiretroviral adherence and CD4+ cell counts in homeless and marginally housed individuals with HIV infection. *Clin Infect Dis*. Jul 15 2006;43(2):234-42.
46. Kushel MB, Perry S, Bangsberg D, Clark R, Moss AR. Emergency department use among the homeless and marginally housed: results from a community-based study. *Am J Public Health*. May 2002;92(5):778-84.
47. Weiser SD, Bangsberg DR, Kegeles S, Ragland K, Kushel MB, Frongillo EA. Food insecurity among homeless and marginally housed individuals living with HIV/AIDS in San Francisco. *AIDS Behav*. Oct 2009;13(5):841-8.

48. Vijayaraghavan M, Penko J, Bangsberg DR, Miaskowski C, Kushel MB. Opioid Analgesic Misuse in a Community-Based Cohort of HIV-Infected Indigent Adults. *JAMA Intern Med*. Feb 11 173(3):235-7. doi:1556794 [pii]
10.1001/jamainternmed.2013.1576
49. California Tobacco Survey. California Tobacco Survey. http://librariesucsdedu/locations/sshl/data-gov-info-gis/ssds/guides/tobacco-surveyshtml.
50. SRNT Subcommittee on Biochemical Verification. Biochemical Verification of tobacco use and cessation. *Nicotine Tob Res*. 2001;4:149-159.
51. Sudore RL, Landefeld CS, Williams BA, Barnes DE, Lindquist K, Schillinger D. Use of a modified informed consent process among vulnerable patients: a descriptive study. *J Gen Intern Med*. Aug 2006;21(8):867-73.
52. Rotheram-Borus MJ, Swendeman D, Comulada WS, Weiss RE, Lee M, Lightfoot M. Prevention for substance-using HIV-positive young people: telephone and in-person delivery. *J Acquir Immune Defic Syndr*. Oct 1 2004;37 Suppl 2:S68-77.
53. Vijayaraghavan M, Penko J, Vittinghoff E, Bangsberg DR, Miaskowski C, Kushel MB. Smoking Behaviors in a Community-Based Cohort of HIV-Infected Indigent Adults. *AIDS Behav*. Aug 6 2013;doi:10.1007/s10461-013-0576-z
54. Hayes RJaM, L.H. Cluster Randomized Trials, Second edition. *Chapman & Hall/CRC Biostatistics Series* 2009;
55. Asian Smokers Quitline. Smoking Cessation Material. *Available at: https://wwwasiansmokersquitlineorg/free_material/; Accessed on February 25, 2022*. 2021;
56. Kick It California. Free Resources to Help you Quit. *Available at: https://kickitcamyshopifycom/collections/all?gf_287531=333526532296%2B333526499528*. Acccessed on October 13, 2022
57. Tong EK, Nguyen TT, Lo P, et al. Lay health educators increase colorectal cancer screening among Hmong Americans: A cluster randomized controlled trial. *Cancer*. Jan 1 2017;123(1):98-106. doi:10.1002/cncr.30265
58. Nguyen TT, Tsoh JY, Woo K, et al. Colorectal Cancer Screening and Chinese Americans: Efficacy of Lay Health Worker Outreach and Print Materials. *American journal of preventive medicine*. Dec 01 2016;doi:10.1016/j.amepre.2016.10.003
59. Jo AM, Nguyen TT, Stewart S, et al. Lay health educators and print materials for the promotion of colorectal cancer screening among Korean Americans: A randomized comparative effectiveness study. *Cancer*. Jul 15 2017;123(14):2705-2715. doi:10.1002/cncr.30568
60. American Dental Hygienists Association and Smoking Cessation Leadership Center. Ask. Advise. Refer. http://wwwaskadvisereferorg/fact_sheetsasp. 2006;
61. Handley MA, Landeros J, Wu C, Najmabadi A, Vargas D, Athavale P. What matters when exploring fidelity when using health IT to reduce disparities? *BMC Med Inform Decis Mak*. Apr 7 2021;21(1):119. doi:10.1186/s12911-021-01476-z
62. Lindson N, Thompson TP, Ferrey A, Lambert JD, Aveyard P. Motivational interviewing for smoking cessation. *Cochrane Database Syst Rev*. Jul 31 2019;7:CD006936. doi:10.1002/14651858.CD006936.pub4
63. Vijayaraghavan M, Freitas D, Bangsberg DR, Miaskowski C, Kushel MB. Non-medical use of non-opioid psychotherapeutic medications in a community-based cohort of HIV-infected indigent adults. *Drug Alcohol Depend*. Oct 1 2014;143:263-7. doi:10.1016/j.drugalcdep.2014.06.044
64. Vijayaraghavan M, Penko J, Guzman D, Miaskowski C, Kushel MB. Primary Care Providers' Judgments of Opioid Analgesic Misuse in a Community-Based Cohort of HIV-Infected Indigent Adults. *J Gen Intern Med*. Nov 9 2010;doi:10.1007/s11606-010-1555-y

65. Miaskowski C, Penko J, Guzman D, Mattson J, Bangsberg DR, Kushel MB. Occurrence and characteristics of chronic pain in a community-based cohort of indigent adults living with HIV infection. *J Pain* 2011;(In Press)
66. Gelberg L, Gallagher T, Andersen R, Koegel P. Competing priorities as a barrier to medical care among homeless adults in Los Angeles. *Am J Public Health*. 1997;87:217-20.
67. Gielen AC, McDonnell KA, Wu AW, O'Campo P, Faden R. Quality of life among women living with HIV: the importance violence, social support, and self care behaviors. *Soc Sci Med*. Jan 2001;52(2):315-22.
68. Tsay SL, Halstead MT, McCrone S. Predictors of coping efficacy, negative moods and post-traumatic stress syndrome following major trauma. *Int J Nurs Pract*. Apr 2001;7(2):74-83.
69. Gelberg L, Andersen RM, Leake BD. The Behavioral Model for Vulnerable Populations: application to medical care use and outcomes for homeless people. *Health Serv Res*. Feb 2000;34(6):1273-302.
70. Vijayaraghavan M PJ, Guzman D, Miaskowski C, Kushel M. Opioid analgesic misuse in a community based cohort of HIV-Infected indigent adults. *In press, Archives of Internal Medicine*.
71. Jaffee KD, Liu GC, Canty-Mitchell J, Qi RA, Austin J, Swigonski N. Race, urban community stressors, and behavioral and emotional problems of children with special health care needs. *Psychiatr Serv*. Jan 2005;56(1):63-9. doi:10.1176/appi.ps.56.1.63
72. Heatherton TF, Kozlowski LT, Frecker RC, Fagerstrom KO. The Fagerstrom Test for Nicotine Dependence: a revision of the Fagerstrom Tolerance Questionnaire. *Br J Addict*. Sep 1991;86(9):1119-27.
73. Strong DR, Pearson J, Ehlke S, et al. Indicators of dependence for different types of tobacco product users: Descriptive findings from Wave 1 (2013-2014) of the Population Assessment of Tobacco and Health (PATH) study. *Drug Alcohol Depend*. Sep 1 2017;178:257-266. doi:10.1016/j.drugalcdep.2017.05.010
74. Halikas JA, Kuhn KL, Crosby R, Carlson G, Crea F. The measurement of craving in cocaine patients using the Minnesota Cocaine Craving Scale. *Compr Psychiatry*. Jan-Feb 1991;32(1):22-7.
75. Patton JH, Stanford MS, Barratt ES. Factor structure of the Barratt impulsiveness scale. *J Clin Psychol*. Nov 1995;51(6):768-74.
76. Al-Delaimy W, Edland S, Pierce JP, Mills AL, White MM. California Tobacco Survey (CTS): 2008. *http://hdlhandlenet/UCSD/10026 California Department of Health Services [Distributor] V4 [Version]*. 2008;
77. National Health Interview Survey (Centers for Disease Control and Prevention) (2017).
78. Klemperer EM, West JC, Peasley-Miklus C, Villanti AC. Change in Tobacco and Electronic Cigarette Use and Motivation to Quit in Response to COVID-19. *Nicotine Tob Res*. Aug 24 2020;22(9):1662-1663. doi:10.1093/ntr/ntaa072
79. Yingst JM, Krebs NM, Bordner CR, Hobkirk AL, Allen SI, Foulds J. Tobacco Use Changes and Perceived Health Risks among Current Tobacco Users during the COVID-19 Pandemic. *Int J Environ Res Public Health*. Feb 12 2021;18(4)doi:10.3390/ijerph18041795
80. Andresen EM, Malmgren JA, Carter WB, Patrick DL. Screening for depression in well older adults: Evaluation of a short form of the CES-D. *American journal of preventive medicine*. 1994;10(2):77-84.
81. Spitzer RL, Kroenke K, Williams JB, Löwe B. A brief measure for assessing generalized anxiety disorder: the GAD-7. *Archives of internal medicine*. 2006;166(10):1092-1097.
82. Prins A, Bovin MJ, Smolenski DJ, et al. The primary care PTSD screen for DSM-5 (PC-PTSD-5): development and evaluation within a veteran primary care sample. *Journal of general internal medicine*. 2016;31(10):1206-1211.

83. WHO Assist Working Group. The Alcohol, Smoking and Substance Involvement Screening Test (ASSIST): development, reliability and feasibility. *Addiction*. Sep 2002;97(9):1183-94.
84. Humeniuk R, Ali R, Babor TF, et al. Validation of the Alcohol, Smoking And Substance Involvement Screening Test (ASSIST). *Addiction*. Jun 2008;103(6):1039-47.
85. Newcombe DA, Humeniuk RE, Ali R. Validation of the World Health Organization Alcohol, Smoking and Substance Involvement Screening Test (ASSIST): report of results from the Australian site. *Drug Alcohol Rev*. May 2005;24(3):217-26.
86. The Alcohol, Smoking and Substance Involvement Screening Test (ASSIST): development, reliability and feasibility. *Addiction*. Sep 2002;97(9):1183-94.
87. Aalto M, Alho H, Halme JT, Seppa K. The Alcohol Use Disorders Identification Test (AUDIT) and its derivatives in screening for heavy drinking among the elderly. *International journal of geriatric psychiatry*. Sep 2011;26(9):881-5. doi:10.1002/gps.2498
88. Bush K, Kivlahan DR, McDonell MB, Fihn SD, Bradley KA. The AUDIT alcohol consumption questions (AUDIT-C): an effective brief screening test for problem drinking. Ambulatory Care Quality Improvement Project (ACQUIP). Alcohol Use Disorders Identification Test. *Archives of internal medicine*. Sep 14 1998;158(16):1789-95.
89. Delucchi KL, Tajima B, Guydish J. Development of the Smoking Knowledge, Attitudes, and Practices (S-KAP) Instrument. *J Drug Issues*. Mar 2009;39(2):347-364.
90. Vijayaraghavan M, Guydish J, Pierce JP. Building Tobacco Cessation Capacity in Homeless Shelters: A Pilot Study. *J Community Health*. Oct 2016;41(5):998-1005. doi:10.1007/s10900-016-0182-x
91. Rubin DB. Inference and missing data. *Biometrika*. 1976;63:581-592.
92. D LRaR. Statistical analysis with missing data (2nd edition) *New York,NY: Wiley* 2002;
93. Schafer J. Analysis of Incomplete Multivariate Data. London: Chapman & Hall. 1997;
94. D'Agostino RB. Propensity score methods for bias reduction in the comparison of a treatment to a non-randomized control group. *Statistics in Medicine*. 1998;17(2265-2281)
95. Rosenbaum PR. The central role of the propensity score in observational studies for causal effects. *Biometrika*. 1983;60:41-55.
96. Rosenbaum PR and DB Rubin. Reducing bias in observations studies using subclassification on the propensity score. *Journal of American Statistical Association*. 1984;(79):516-524.
